# Genome-Wide Computational Prediction and Analysis of Core Promoter Elements across Plant Monocots and Dicots

**DOI:** 10.1371/journal.pone.0079011

**Published:** 2013-10-29

**Authors:** Sunita Kumari, Doreen Ware

**Affiliations:** 1 Cold Spring Harbor Laboratory, Cold Spring Harbor, New York, United States of America,; 2 United States Department of Agriculture-Agriculture Research Service, Robert W. Holley Center for Agriculture and Health, Ithaca, New York, United States of America; Michigan State University, United States of America

## Abstract

Transcription initiation, essential to gene expression regulation, involves recruitment of basal transcription factors to the core promoter elements (CPEs). The distribution of currently known CPEs across plant genomes is largely unknown. This is the first large scale genome-wide report on the computational prediction of CPEs across eight plant genomes to help better understand the transcription initiation complex assembly. The distribution of thirteen known CPEs across four monocots (*Brachypodium distachyon, Oryza sativa ssp. japonica, Sorghum bicolor, Zea mays*) and four dicots (*Arabidopsis thaliana, Populus trichocarpa, Vitis vinifera, Glycine max*) reveals the structural organization of the core promoter in relation to the TATA-box as well as with respect to other CPEs. The distribution of known CPE motifs with respect to transcription start site (TSS) exhibited positional conservation within monocots and dicots with slight differences across all eight genomes. Further, a more refined subset of annotated genes based on orthologs of the model monocot (*O. sativa* ssp. *japonica*) and dicot (*A. thaliana*) genomes supported the positional distribution of these thirteen known CPEs. DNA free energy profiles provided evidence that the structural properties of promoter regions are distinctly different from that of the non-regulatory genome sequence. It also showed that monocot core promoters have lower DNA free energy than dicot core promoters. The comparison of monocot and dicot promoter sequences highlights both the similarities and differences in the core promoter architecture irrespective of the species-specific nucleotide bias. This study will be useful for future work related to genome annotation projects and can inspire research efforts aimed to better understand regulatory mechanisms of transcription.

## Introduction

Despite numerous technological advances in biological and computational sciences in the post- genome era, our basic understanding of gene regulatory mechanisms remains primitive. Currently, the fundamental need to understand RNA polymerase II (polII) mediated transcription initiation is well recognized for developing system level understanding of the condition-specific gene regulatory networks (GRNs). It is now well known that the TATA-box motif, once thought to be necessary for formation of polII pre-initiation complex (PIC) assembly, only accounts for a small fraction of the expressed genome [1], [2], [3]. Furthermore, it is still challenging to accurately identify the transcription start site (TSS) and predict the functional genomic elements in the promoter region. Therefore, incorporation of TSS and cis-regulatory element identification tools into genome annotation pipelines has yet to become a common practice. While experimental approaches like yeast-1-hybrid (Y1H) [4] and chromatin-immunoprecipitation (ChIP) assays [5] have made great strides in identifying transcription factor binding sites (TFBS) for a few model organisms, there are still technical and cost barriers to implement these methods on a large scale. There is a need for robust bioinformatics methods that can accurately identify TSS and predict the TFBS for the plant genomes. Reliable prediction of core promoter elements holds the promise to bridge this gap.

Promoter regions can be categorized into two classes: core (proximal) and extended (distal). The core promoter is the primary docking site of polII PIC and directs basal transcription [6], [7]. The cis-regulatory elements in the extended promoter region are thought to control spatial and temporal expression of their associated gene(s) [8]. Transcription of protein coding genes depends on the formation of the PIC assembly that includes RNA polymerase II, the general transcription factors (TFIIB, TFIID, TFIIE, TFIIF, TFIIH) along with co-activators and other protein complexes [9], [10]. A subunit of transcription factor TFIID complex, the TATA-binding protein (TBP), binds to the TATA-box, which is located ∼30 bp upstream of the TSS and nucleates PIC assembly [11], [12]. However, only 13% of yeast promoters and 10% of human promoters contain the TATA-box [13]. In Arabidopsis, around 29% of promoters have been reported to contain a TATA-box, ∼32 bp upstream with respect to the TSS [14] whereas in rice around 19% of promoters contain TATA-box [15]. Previous studies in yeast and human have reported that the TATA-box is generally associated with tissue specific expression and mostly regulated by stress stimuli whereas TATA-less genes are constitutively expressed and predominantly involved in housekeeping processes [13], [16], [17], [18]. These findings suggest that core promoter architecture has a strong influence on the transcriptional regulation. As TATA-containing promoters are far less prevalent than TATA-less promoters, other DNA-elements must be responsible for coordinating transcription in a sizeable number of promoters. In *Saccharomyces cerevisiae*, *Drosophila melanogaster*, and mammals, several other CPEs have been identified in TATA and TATA-less promoters that include the initiator (Inr) element located at or surrounding the TSS which is recognized by TAF1 and TAF2 subunits of the TFIID complex [14], the TFIIB recognition element (BRE) located immediately upstream (BREu) [19] and/or downstream (BREd) of the TATA-box [20], the downstream promoter element (DPE) located between positions +28 to +33 (relative to the TSS) which is recognized by TAF6 and TAF9 subunits of the TFIID complex [21], the motif ten element (MTE) located between positions +18 and +29 [22], and the downstream core element (DCE) located around positions +6 to +35 [23]. Other less characterized downstream elements include the X gene Core Promoter Element 1 (XCPE1) located between positions −8 to +2 in hepatitis B virus X gene promoter and found in ∼1% of human TATA-less genes [24], and the Multiple start site Element Downstream (MED-1) identified in the majority of TATA-less promoters of mammalian genes analyzed [25]. The CCAAT-box is located between −300 to −80 bp from TSS in human promoters [26] and there is evidence for conservation of this element in other eukaryotes including plants [27], [28], [29]. Based on a further comparison of CPEs of mammalian and plants, CpG islands were found mainly in mammalian promoters whereas the Y-patch (also called the pyrimidine patch) was found to be plant specific [30].

Very little is known about the cis-regulatory elements of transcription control in plants. In the past decade, considerable work has focused on model animal species like *D. melanogaster* [1], [31], [32], [33], [34], [35], [36], [37], [38], *Caenorhabditis elegans* [39], [40], [41], *Rattus norvegicus* [42], [43], [44], [45], [46], [47], [48], *Mus musculus* [49], [50], [51], [52], [53], [54], [55], [56], [57], [58], [59], [60], [61], and *Homo sapiens* [2], [31], [51], [60], [62], [63], [64], [65], [66], [67], [68], [69], [70], [71], [72], [73], [74]. However, plant core promoters have yet to be thoroughly analyzed [75]. Previous studies in plants have focused mainly on Arabidopsis and rice and generally included the identification of TATA-box and Y-patch elements in selected sets of promoter sequences [14], [15]. It was reported that ∼ 50% of rice gene promoters possess one or more Y-patches in their core promoters [15]. The plant promoter database (ppdb) [76] also identifies putative TATA-box and Y-patch elements in several plant genomes using the computational method of local distribution of short sequences (LDSS) [77]. Cis-regulatory regions have been predicted based on the free energy of DNA melting in Arabidopsis and rice genomes [78]. The packaging of DNA into chromatin, DNA methylation, and chromatin structure demonstrates the uniqueness in the promoter structure and create multiple levels of complexity to the regulation of gene expression [79], [80]. Thus, the identification of CPEs is essential in understanding the logic behind transcriptional regulation [30], [81], [82]. Analysis of CPEs at the whole genome level in different plant genomes will contribute to fundamental insights into the mechanisms by which transcription occurs in plants and how it differs from other eukaryotes.

Due to the complexity, diversity and inherent degenerate nature of regulatory motifs within promoters, the prediction of cis-regulatory elements is quite challenging and *in silico* prediction is still in its early stage. Though the number of computational motif discovery methods has significantly increased in last two decades [83], there is no single method that adequately captures all types of regulatory motif patterns [84], [85], [86], [87]. Existing promoter analysis tools cannot reliably identify cis regulatory elements in a genomic sequence, thus predicting too many false positives because these tools are generally focused only on the sequence content [88]. Position weight matrices (PWMs) use the log-likelihood scoring function for computing a match score for potential binding sites and therefore have been reported to be better measure than the consensus sequence [89], [90]. However, it is still challenging for PWM based predictive methods to distinguish functional TFBS from non-functional predictions without applying additional refinements such as cross-species conservation [91], [92], [93]. Functional studies on understanding the role of conserved genomic regions from species to species have shown positional conservation to be one of the key biological characteristics of the DNA-motifs in a regulatory context [94], [95], [96]. Therefore, predictions of TFBS with respect to TSS of orthologous genes are expected to reduce false positive rates and might be potentially functional. Our study leveraged monocot and dicot orthologous genes to provide additional metric for giving higher confidence to the TFBS prediction results that we believe to be testable for biological relevance. Recently, DNA free energy profiles have also been used for predicting TSS that significantly improved the motif discovery in yeast [81], [97].

We performed a genome-wide prediction of known CPEs in eight plant species spanning both monocots and dicots, by developing a systematic and unbiased high-throughput methodology using PWMs, DNA free energy profiles, and homology to significantly reduce the false positive rate of motif discovery. The CPE profiles were compared to see the similarities and differences in promoter sequence architecture within and across monocots and dicots.

## Results

Core promoter regions are generally reported within a tight window of TSS±50bases [2], [14], [98]. Even though each CPE's reported motif signal position in this region is strong and likely represents the binding site location ultimately responsible for polII assembly in eukaryotes [1], [35], [99], [100], it is not known if this represents local or global maxima in the CPE's relative abundance with respect to TSS. Experimental studies of promoter structure and function have reported high core promoter activity in regions that are on average 300bases upstream of TSS [2]. This led us to broaden the search space to TSS±500bases to produce a more comprehensive frequency profile for each CPE.

To predict CPEs, the promoter sequences of protein coding genes in eight plant genomes were extracted from the Gramene core databases (version 34b) [www.gramene.org]. These eight plant genomes included four monocots (*Brachypodium distachyon* (Bdi), *Oryza sativa ssp. japonica* (Osa), *Sorghum bicolor* (Sbi), *Zea mays* (Zma)) and four dicots (*Arabidopsis thaliana* (Ath), *Populus trichocarpa* (Ptr), *Vitis vinifera* (Vvi), *Glycine max* (Gma)). For each genome, only the transcripts annotated with a 5′ untranslated region (5′UTR) and high quality filtered gene-set (after discarding transposable elements) were used for CPEs predictions (Table S1). Among dicots 77% Ath, 67% Gma, 59% Ptr, and 57% Vvi whereas among monocots 37% Bdi, 65% Osa, 36% Sbi, and 73% Zma coding transcripts were found to have 5′UTR annotations (Table S1). The distribution of the number of transcripts with respect to 5′UTR length in dicots and monocots is shown in Figure S1.

We selected only those CPEs for this study that had PWM information publically available. These included TATA-box, Initiator element (Inr) and CCAAT-box from PlantProm database [101]. In PlantProm, Inr element is also referred as TSS [101]. Other CPEs included TFIIB Recognition Elements BREu and BREd, GC-box, X-Core Promoter Element (XCPE1), Multiple start site Element Downstream (MED-1), Motif-Ten element (MTE), Downstream Core Element (DCE-S1, DCE-S2, DCE-S3), and Downstream Promoter Element (DPE) from the JASPAR POLII database [102]; and pyrimidine patch (Y-patch) from literature [15]. A brief description of the CPEs with PWM logo is given in Table S2. For each motif, CPE prediction results were filtered based on motif specific prediction score cut-off, given in Table S3 (for details, see methods).

### Experimental Design

**Figure 1:**
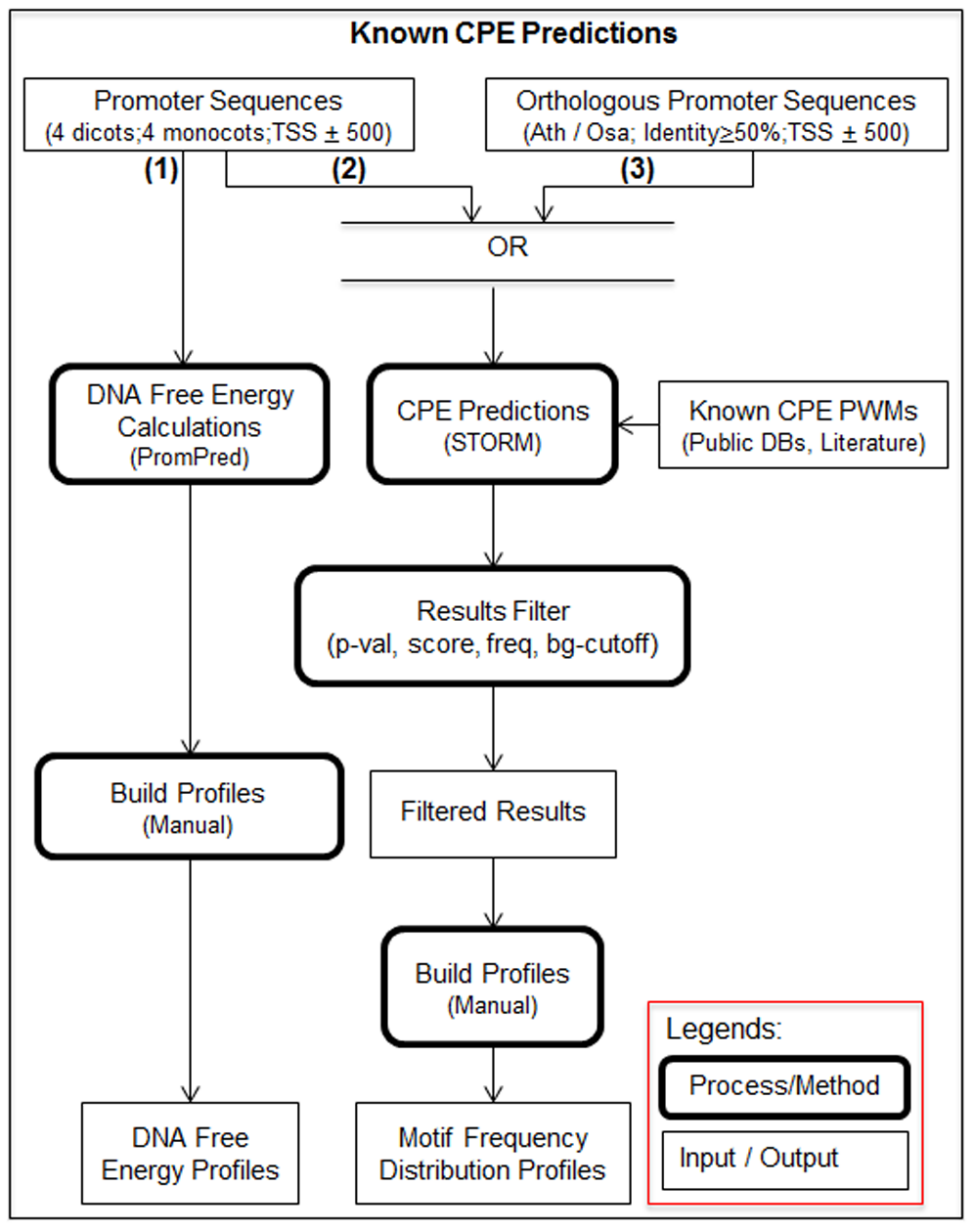
Flow diagram of computational prediction of known core promoter elements in eight plant genomes. Methods included the use of DNA free energy profiles and prediction of known CPE types using published PWM profiles. Overrepresentation near TSS and conservation of putative sites among orthologous genes within dicot and monocot groups were used as filters to increase the confidence of CPE calls.

The flow diagram for genome-wide computational prediction of known CPEs are given in Figure 1. It shows the prediction of known core promoter elements in the eight plant genomes using three approaches. First, DNA free energy profiles of the promoter region were studied to detect differences in the structural properties of DNA across monocots and dicots. It also differentiated regulatory from non-regulatory regions and helped in delineating the boundaries of the regulatory region. Second, predefined PWMs were used to locate putative CPEs that are overrepresented in a 1000 bp window centered on the TSS. Third, predictions based on orthologous promoter sequences were used as an additional metric to select and increase the confidence of putative CPEs identified in the previous step. *Arabidopsis thaliana* and *Oryza sativa ssp. japonica* were used as the model dicot and monocot genomes respectively for homology-based CPE predictions. CPE predictions were further filtered based on a motif-specific prediction score cut-off, frequency of a CPE-motif occurrence in a sequence, and foreground CPE-motif signal cutoff based on CPE-motif signal observed in the background genomic sequences. These predictions were used to build frequency distribution profiles for each PWM for each plant genome (see methods for details). PromPredict [103], was used for building DNA free energy profiles; whereas Search Tool for Occurrences of Regulatory Motifs (STORM) [60], [104], [105] was used for identifying each known CPE pattern (encoded as a PWM) in the core promoter sequences.

To assess the reliability of our prediction methodology and to tune our prediction pipeline, we also examined the above mentioned known CPEs using experimentally derived set of promoter elements of *Drosophila melanogaster* from Eukaryotic promoter database [106] and compared the predictions of selected CPEs that have been experimentally confirmed and reported in literature [22], [35], [107].

### Delineation of core promoter region using DNA free energy profiles

**Figure 2:**
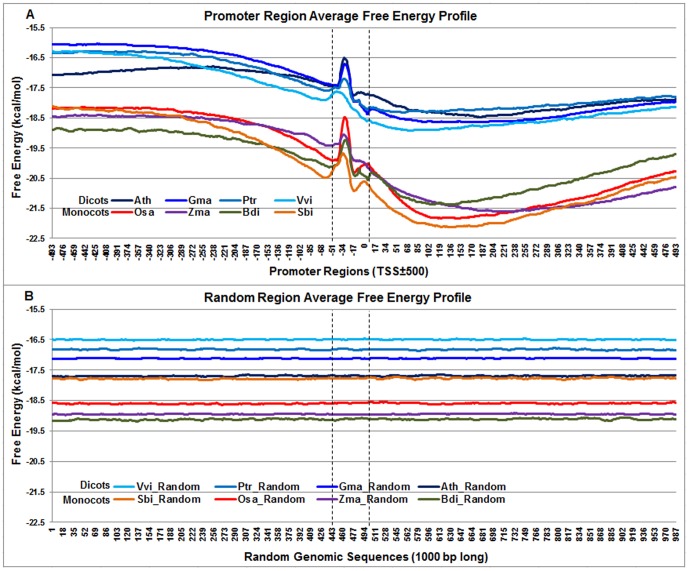
Genome-wide distribution of promoter-region DNA free energy profiles across eight plant genomes. Panel A: DNA free energy distribution profile of the core promoter-region across four dicots -*Arabidopsis thaliana* (Ath - solid navy blue), *Glycine max* (Gma-solid dark blue), *Populus trichocarpa* (Ptr –solid blue sapphire), and *Vitis vinifera* (Vvi -solid blue green) and four monocots - *Brachypodium distachyon* (Bdi-solid bronze yellow), *Oryza sativa ssp. japonica* (Osa-solid red), *Sorghum bicolor* (Sbi-solid bronze), and *Zea mays* (Zma -solid purple). The dicots showed higher average free energy than the monocots and their free energy profiles were distinctly separated from monocot energy profiles. The core promoter region between two vertical dotted black lines (around TSS) shows a sharp peak of instability near the TSS, against an overall trend of increasing stability going from upstream to downstream of the TSS. Panel B: The DNA free energy distribution of random 1000-bp windows of genomic sequence for the same species, showing flat profiles.

Based on the differences in the average DNA free energy profiles, regulatory regions can be discriminated from non-regulatory regions and start sites of transcription can be approximated [78]. DNA free energy profiles were generated for promoter regions [TSS±500] as foreground and non-promoter regions [randomly selected 1000 bp long windows] as background in eight genomes using PromPredict [103](Figure 2). The free energy of DNA melting depends on the base stacking energy of dinucleotide sequences and on GC content. As shown in Figure S2, monocot transcripts have distinctly higher GC content (50–65%) than dicots (35–45%). In addition, the average free energy values for the upstream and downstream region with respect to TSS are different depending on the GC content of the region in each genome. The free energy profiles were obtained by averaging DNA free energy of each base across all promoter sequences and were distinctly segregated into monocot and dicot specific clusters (Figure 2A). On average, regulatory regions in the monocots had lower DNA free energy (−20 kcal/mol±0.14 SD) as compared to dicots (−17.6 kcal/mol±0.15 SD). However the shape of the regulatory genome energy profile across all eight genomes was remarkably consistent and distinctly different from the shape of non-regulatory genome energy profile (Figure 2B). The energy profile of non-regulatory genomic regions across all genomes, though consistently flat, had a nearly five-fold higher standard deviation as compared to the regulatory genome (monocots averaged −18.5 kcal/mol±0.68 and dicots averaged −16.8 kcal/mol±0.77). The energy profile around the TSS [−100 to +50 with respect to the TSS] goes through at least two distinct local minima and two distinct local maxima with Vvi being an exception with only one distinct local minima and one distinct local maxima. This entire window of 150bases around the TSS exhibits a tight and significantly alternating pattern in DNA structure stability and instability, thus making it a putative hotspot of polII assembly and transcription initiation. Based on these observations, it is reasonable to think that the free energy profile in this window of ∼150bases defines the characteristic signature of a core regulatory region that distinguishes it from non-regulatory regions. This region, taken together with the energy profile of its flanking regions, further helps in demarcating the boundaries of the regulatory region.

### Computational prediction of CPEs based on the positional and orthologous gene conservation across genomes

**Figure 3:**
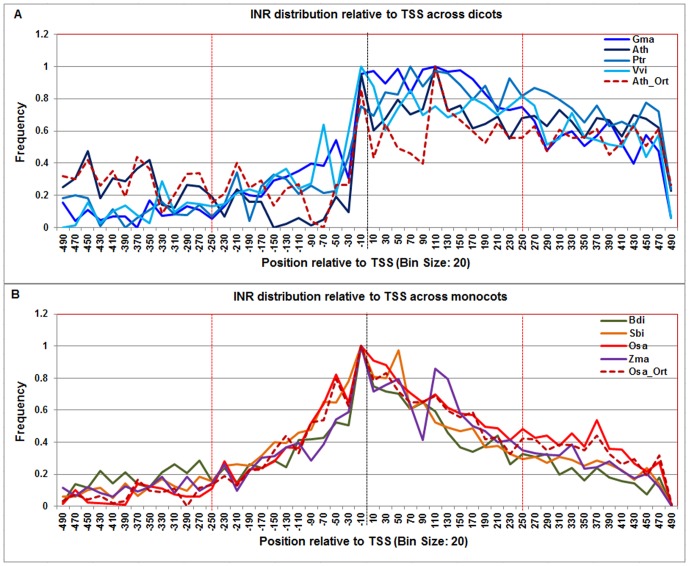
Normalized frequency distribution profile of Inr with respect to TSS across monocots and dicots. Panel A: Genome-wide positional distribution of Inr frequency profile across four dicots: *Arabidopsis thaliana* (Ath - solid navy blue), *Glycine max* (Gma-solid dark blue), *Populus trichocarpa* (Ptr –solid blue sapphire), and *Vitis vinifera* (Vvi -solid blue green). Genome-wide functional distribution profile of Inr based on the ortholog mapping of Ath with rest of the dicots (Ath:Gma, Ath:Ptr, and Ath:Vvi) is shown in dotted brown colored line. Panel B: Genome-wide positional distribution of Inr element frequency profile across four monocots: *Brachypodium distachyon* (Bdi-solid bronze yellow), *Oryza sativa ssp. japonica* (Osa-solid red), *Sorghum bicolor* (Sbi-solid bronze), and *Zea mays* (Zma -solid purple). Genome-wide functional distribution profile of Inr based on the ortholog mapping of Osa with rest of the monocots (Osa:Bdi, Osa:Sbi, and Osa:Zma) is shown in dotted brown colored line. X-axis shows [−500,+500 with respect to TSS] that is binned into 20 base-pair bins, where each bin is represented by the bin-center. Y-axis shows the normalized frequency distribution of the Inr element.

**Figure 4:**
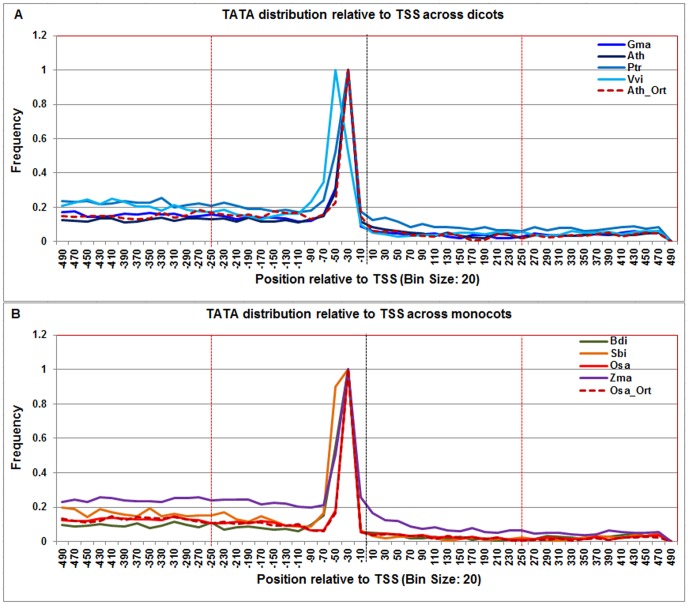
Normalized frequency distribution profile of TATA-box with respect to TSS across monocots and dicots. Panel A: Genome-wide positional distribution of TATA-box frequency profile across four dicots: *Arabidopsis thaliana* (Ath - solid navy blue), *Glycine max* (Gma-solid dark blue), *Populus trichocarpa* (Ptr –solid blue sapphire), and *Vitis vinifera* (Vvi -solid blue green). Genome-wide functional distribution profile of TATA-box based on the ortholog mapping of Ath with rest of the dicots (Ath:Gma, Ath:Ptr, and Ath:Vvi) is shown in dotted brown colored line. Panel B: Genome-wide positional distribution of TATA-box element frequency profile across four monocots: *Brachypodium distachyon* (Bdi-solid bronze yellow), *Oryza sativa ssp. japonica* (Osa-solid red), *Sorghum bicolor* (Sbi-solid bronze), and *Zea mays* (Zma -solid purple). Genome-wide functional distribution profile of TATA-box based on the ortholog mapping of Osa with rest of the monocots (Osa:Bdi, Osa:Sbi, and Osa:Zma) is shown in dotted brown colored line. X-axis shows [−500,+500 with respect to TSS] that is binned into 20 base-pair bins, where each bin is represented by the bin-center. Y-axis shows the normalized frequency distribution of the TATA-box element.

**Figure 5:**
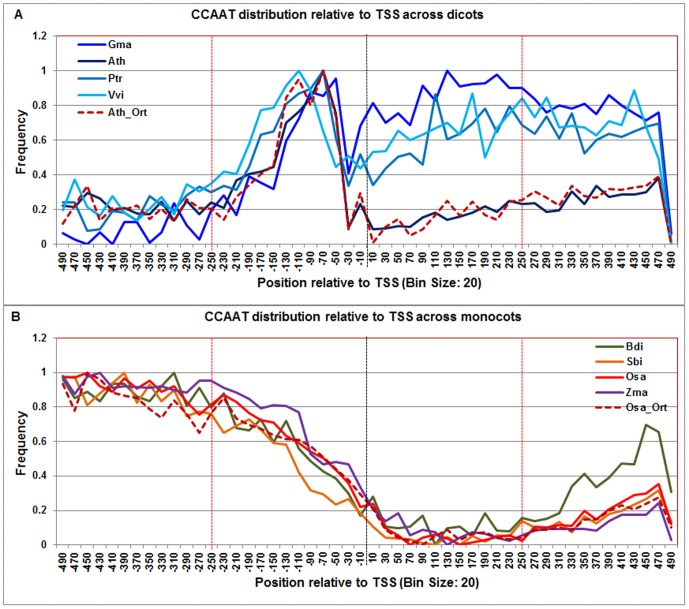
Normalized frequency distribution profile of CCAAT-box with respect to TSS across monocots and dicots. Panel A: Genome-wide positional distribution of CCAAT-box frequency profile across four dicots: *Arabidopsis thaliana* (Ath - solid navy blue), *Glycine max* (Gma-solid dark blue), *Populus trichocarpa* (Ptr –solid blue sapphire), and *Vitis vinifera* (Vvi -solid blue green). Genome-wide functional distribution profile of CCAAT-box based on the ortholog mapping of Ath with rest of the dicots (Ath:Gma, Ath:Ptr, and Ath:Vvi) is shown in dotted brown colored line. Panel B: Genome-wide positional distribution of CCAAT-box element frequency profile across four monocots: *Brachypodium distachyon* (Bdi-solid bronze yellow), *Oryza sativa ssp. japonica* (Osa-solid red), *Sorghum bicolor* (Sbi-solid bronze), and *Zea mays* (Zma-solid purple). Genome-wide functional distribution profile of CCAAT-box based on the ortholog mapping of Osa with rest of the monocots (Osa:Bdi, Osa:Sbi, and Osa:Zma) is shown in dotted brown colored line. X-axis shows [−500,+500 with respect to TSS] that is binned into 20 base-pair bins, where each bin is represented by the bin-center. Y-axis shows the normalized frequency distribution of the CCAAT-box element.

**Figure 6:**
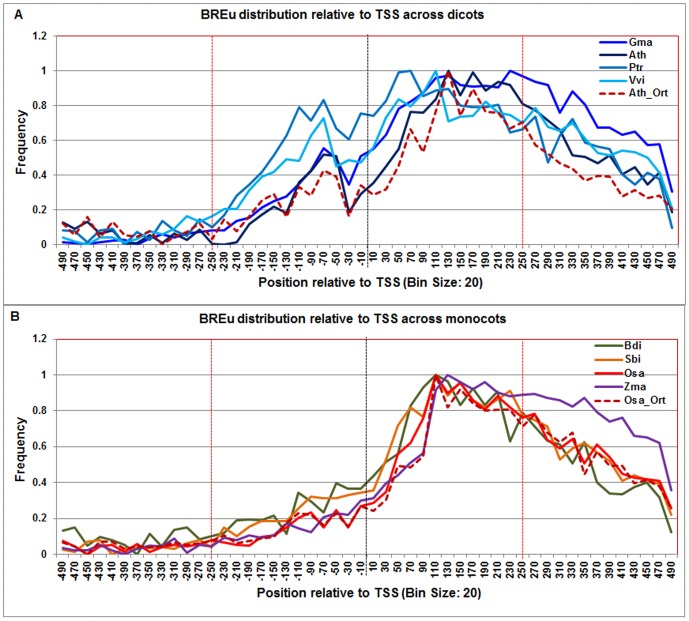
Normalized frequency distribution profile of BREu with respect to TSS across monocots and dicots. Panel A: Genome-wide positional distribution of BREu element frequency profile across four dicots: *Arabidopsis thaliana* (Ath -solid navy blue), *Glycine max* (Gma-solid dark blue), *Populus trichocarpa* (Ptr –solid blue sapphire), and *Vitis vinifera* (Vvi -solid blue green). Genome-wide functional distribution profile of BREu based on the ortholog mapping of Ath with rest of the dicots (Ath:Gma, Ath:Ptr, and Ath:Vvi) is shown in dotted brown colored line. Panel B: Genome-wide positional distribution of BREu element frequency profile across four monocots: *Brachypodium distachyon* (Bdi-solid bronze yellow), *Oryza sativa ssp. japonica* (Osa-solid red), *Sorghum bicolor* (Sbi-solid bronze), and *Zea mays* (Zma -solid purple). Genome-wide functional distribution profile of BREu based on the ortholog mapping of Osa with rest of the monocots (Osa:Bdi, Osa:Sbi, and Osa:Zma) is shown in dotted brown colored line. X-axis shows [−500,+500 with respect to TSS] that is binned into 20 base-pair bins, where each bin is represented by the bin-center. Y-axis shows the normalized frequency distribution of the BREu element.

**Figure 7:**
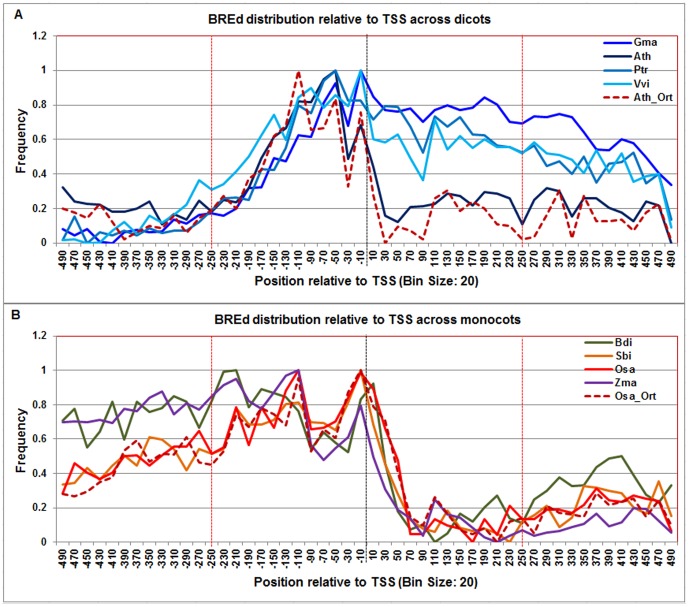
Normalized frequency distribution profile of BREd with respect to TSS across monocots and dicots. Panel A: Genome-wide positional distribution of BREd element frequency profile across four dicots: *Arabidopsis thaliana* (Ath - solid navy blue), *Glycine max* (Gma-solid dark blue), *Populus trichocarpa* (Ptr –solid blue sapphire), and *Vitis vinifera* (Vvi -solid blue green). Genome-wide functional distribution profile of BREd based on the ortholog mapping of Ath with rest of the dicots (Ath:Gma, Ath:Ptr, and Ath:Vvi) is shown in dotted brown colored line. Panel B: Genome-wide positional distribution of BREd element frequency profile across four monocots: *Brachypodium distachyon* (Bdi-solid bronze yellow), *Oryza sativa ssp. japonica* (Osa-solid red), *Sorghum bicolor* (Sbi-solid bronze), and *Zea mays* (Zma -solid purple). Genome-wide functional distribution profile of BREd based on the ortholog mapping of Osa with rest of the monocots (Osa:Bdi, Osa:Sbi, and Osa:Zma) is shown in dotted brown colored line. X-axis shows [−500,+500 with respect to TSS] that is binned into 20 base-pair bins, where each bin is represented by the bin-center. Y-axis shows the normalized frequency distribution of the BREd element.

**Figure 8:**
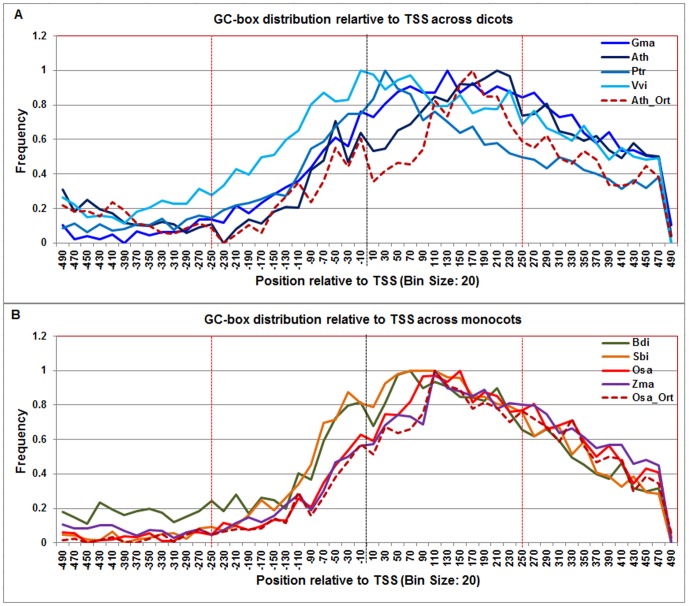
Normalized frequency distribution profile of GC-box with respect to TSS across monocots and dicots. Panel A: Genome-wide positional distribution of GC-box frequency profile across four dicots: *Arabidopsis thaliana* (Ath - solid navy blue), *Glycine max* (Gma-solid dark blue), *Populus trichocarpa* (Ptr –solid blue sapphire), and *Vitis vinifera* (Vvi -solid blue green) is shown. Genome-wide functional distribution profile of GC-box based on the ortholog mapping of Ath with rest of the dicots (Ath:Gma, Ath:Ptr, and Ath:Vvi) is shown in dotted brown colored line. Panel B: Genome-wide positional distribution of GC-box element frequency profile across four monocots: *Brachypodium distachyon* (Bdi-solid bronze yellow), *Oryza sativa ssp. japonica* (Osa-solid red), *Sorghum bicolor* (Sbi-solid bronze), and *Zea mays* (Zma -solid purple). Genome-wide functional distribution profile of GC-box based on the ortholog mapping of Osa with rest of the monocots (Osa:Bdi, Osa:Sbi, and Osa:Zma) is shown in dotted brown colored line. X-axis shows [−500,+500 with respect to TSS] that is binned into 20 base-pair bins, where each bin is represented by the bin-center. Y-axis shows the normalized frequency distribution of the GC-box element.

**Figure 9:**
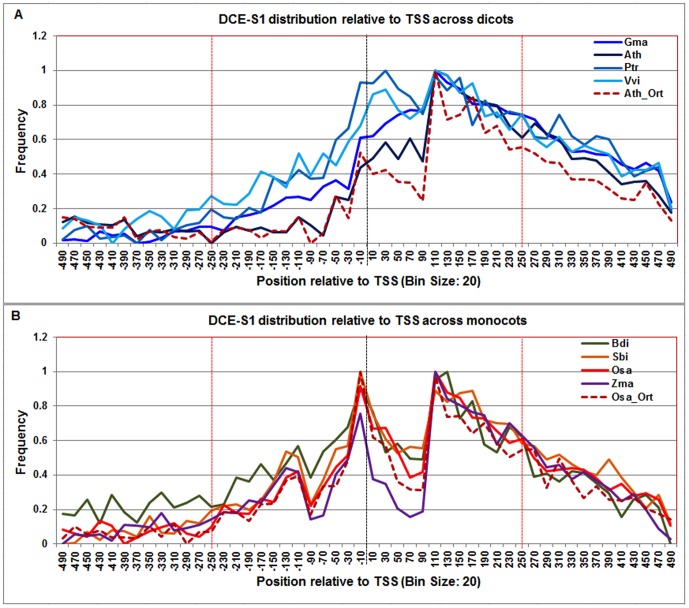
Normalized frequency distribution profile of DCE-S1 with respect to TSS across monocots and dicots. Panel A: Genome-wide positional distribution of DCE-S1 frequency profile across four monocots: *Arabidopsis thaliana* (Ath - solid navy blue), *Glycine max* (Gma-solid dark blue), *Populus trichocarpa* (Ptr –solid blue sapphire), and *Vitis vinifera* (Vvi -solid blue green). Genome-wide functional distribution profile of DCE-S1 based on the ortholog mapping of Ath with rest of the dicots (Ath:Gma, Ath:Ptr, and Ath:Vvi) is shown in dotted brown colored line. Panel B: Genome-wide positional distribution of DCE-S1frequency profile across four monocots: *Brachypodium distachyon* (Bdi-solid bronze yellow), *Oryza sativa ssp. japonica* (Osa-solid red), *Sorghum bicolor* (Sbi-solid bronze), and *Zea mays* (Zma -solid purple). Genome-wide functional distribution profile of DCE-S1 based on the ortholog mapping of Osa with rest of the monocots (Osa:Bdi, Osa:Sbi, and Osa:Zma) is shown in dotted brown colored line. X-axis shows [−500,+500 with respect to TSS] that is binned into 20 base-pair bins, where each bin is represented by the bin-center. Y-axis shows the normalized frequency distribution of the DCE-S1 element.

**Figure 10:**
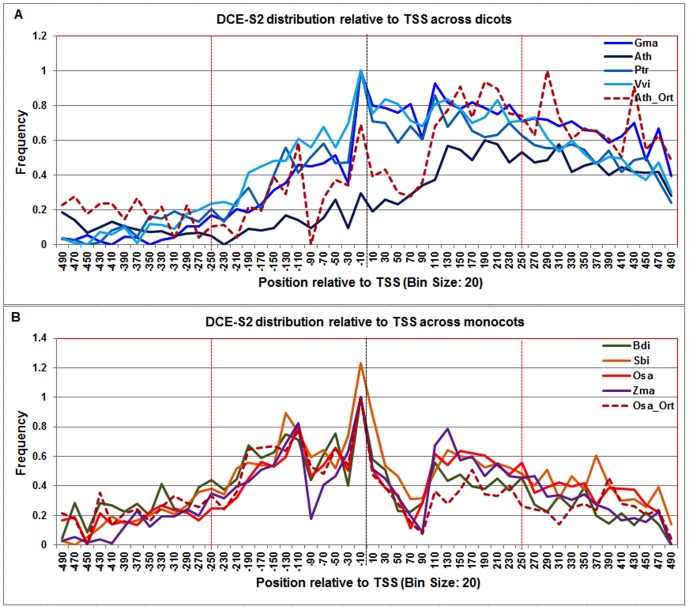
Normalized frequency distribution profile of DCE-S2 with respect to TSS across monocots and dicots. Panel A: Genome-wide positional distribution of DCE-S2 frequency profile across four dicots: *Arabidopsis thaliana* (Ath-solid navy blue), *Glycine max* (Gma-solid dark blue), *Populus trichocarpa* (Ptr –solid blue sapphire), and *Vitis vinifera* (Vvi -solid blue green). Genome-wide functional distribution profile of DCE-S2 based on the ortholog mapping of Ath with rest of the dicots (Ath:Gma, Ath:Ptr, and Ath:Vvi) is shown in dotted brown colored line. Panel B: Genome-wide positional distribution of DCE-S2 element frequency profile across four monocots: *Brachypodium distachyon* (Bdi-solid bronze yellow), *Oryza sativa ssp. japonica* (Osa-solid red), *Sorghum bicolor* (Sbi-solid bronze), and *Zea mays* (Zma -solid purple). Genome-wide functional distribution profile of DCE-S2 based on the ortholog mapping of Osa with rest of the monocots (Osa:Bdi, Osa:Sbi, and Osa:Zma) is shown in dotted brown colored line. X-axis shows [−500,+500 with respect to TSS] that is binned into 20 base-pair bins, where each bin is represented by the bin-center. Y-axis shows the normalized frequency distribution of the DCE-S2 element.

**Figure 11:**
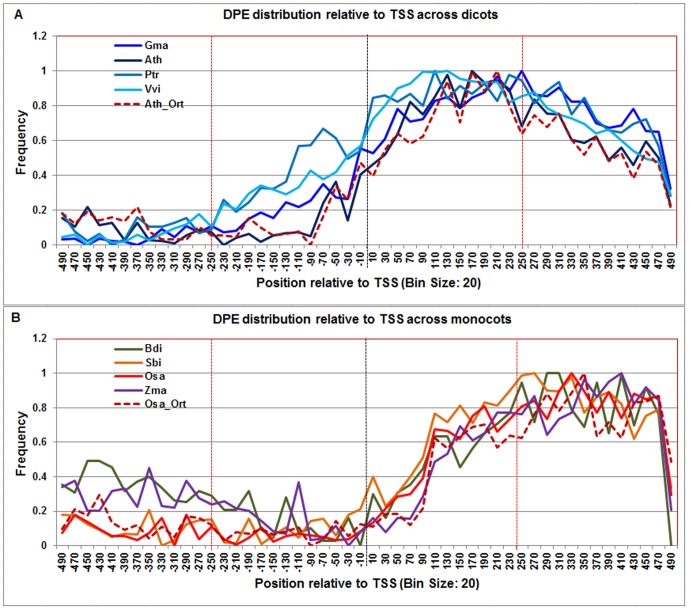
Normalized frequency distribution profile of DPE with respect to TSS across monocots and dicots. Panel A: Genome-wide positional distribution of DPE frequency profile across four dicots: *Arabidopsis thaliana* (Ath-solid navy blue), *Glycine max* (Gma-solid dark blue), *Populus trichocarpa* (Ptr –solid blue sapphire), and *Vitis vinifera* (Vvi-solid blue green). Genome-wide functional distribution profile of DPE based on the ortholog mapping of Ath with rest of the dicots (Ath:Gma, Ath:Ptr, and Ath:Vvi) is shown in dotted brown colored line. Panel B: Genome-wide positional distribution of DPE frequency profile across four monocots: *Brachypodium distachyon* (Bdi-solid bronze yellow), *Oryza sativa ssp. japonica* (Osa-solid red), *Sorghum bicolor* (Sbi-solid bronze), and *Zea mays* (Zma -solid purple). Genome-wide functional distribution profile of DPE based on the ortholog mapping of Osa with rest of the monocots (Osa:Bdi, Osa:Sbi, and Osa:Zma) is shown in dotted brown colored line. X-axis shows [−500,+500 with respect to TSS] that is binned into 20 base-pair bins, where each bin is represented by the bin-center. Y-axis shows the normalized frequency distribution of the DPE.

**Figure 12:**
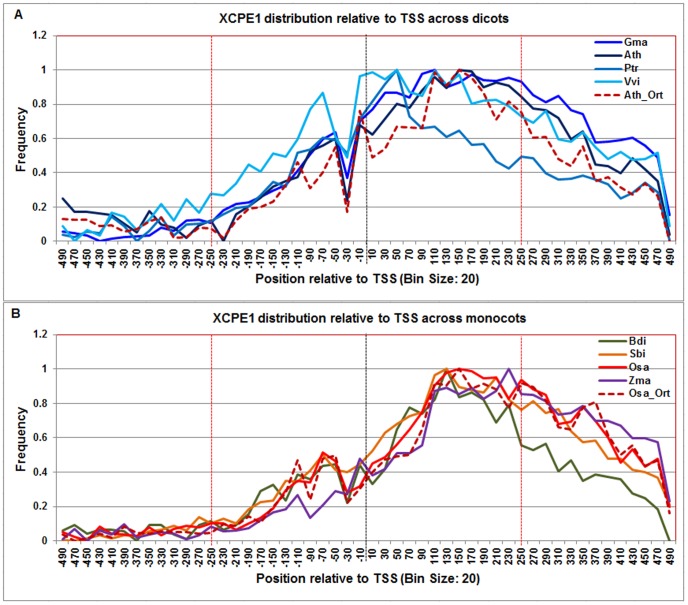
Normalized frequency distribution profile of XCPE1 with respect to TSS across monocots and dicots. Panel A: Genome-wide positional distribution of XCPE1 element frequency profile across four dicots: *Arabidopsis thaliana* (Ath-solid navy blue), *Glycine max* (Gma-solid dark blue), *Populus trichocarpa* (Ptr –solid blue sapphire), and *Vitis vinifera* (Vvi -solid blue green) is shown. Genome-wide functional distribution profile of XCPE1 based on the ortholog mapping of Ath with rest of the dicots (Ath:Gma, Ath:Ptr, and Ath:Vvi) is shown in dotted brown colored line. Panel B: Genome-wide positional distribution of XCPE1 element frequency profile across four monocots: *Brachypodium distachyon* (Bdi-solid bronze yellow), *Oryza sativa ssp. japonica* (Osa-solid red), *Sorghum bicolor* (Sbi-solid bronze), and *Zea mays* (Zma -solid purple). Genome-wide functional distribution profile of XCPE1 based on the ortholog mapping of Osa with rest of the monocots (Osa:Bdi, Osa:Sbi, and Osa:Zma) is shown in dotted brown colored line. X-axis shows [−500,+500 with respect to TSS] that is binned into 20 base-pair bins, where each bin is represented by the bin-center. Y-axis shows the normalized frequency distribution of the XCPE1 element.

**Figure 13:**
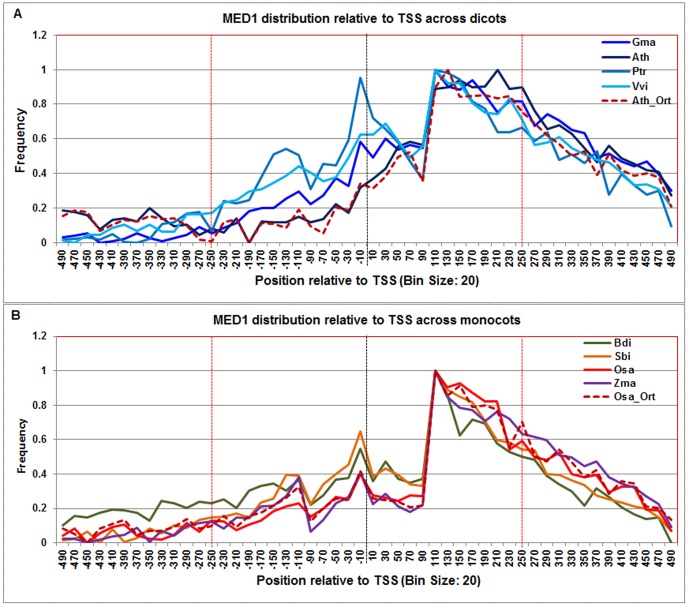
Normalized frequency distribution profile of MED-1 with respect to TSS across monocots and dicots. Panel A: Genome-wide positional distribution of MED-1 frequency profile across four dicots: *Arabidopsis thaliana* (Ath - solid navy blue), *Glycine max* (Gma-solid dark blue), *Populus trichocarpa* (Ptr –solid blue sapphire), and *Vitis vinifera* (Vvi -solid blue green) is shown. Genome-wide functional distribution profile of MED-1 based on the ortholog mapping of Ath with rest of the dicots (Ath:Gma, Ath:Ptr, and Ath:Vvi) is shown in dotted brown colored line. Panel B: Genome-wide positional distribution of MED-1 element frequency profile across four monocots: *Brachypodium distachyon* (Bdi-solid bronze yellow), *Oryza sativa ssp. japonica* (Osa-solid red), *Sorghum bicolor* (Sbi-solid bronze), and *Zea mays* (Zma -solid purple). Genome-wide functional distribution profile of MED-1 based on the ortholog mapping of Osa with rest of the monocots (Osa:Bdi, Osa:Sbi, and Osa:Zma) is shown in dotted brown colored line. X-axis shows [−500,+500 with respect to TSS] that is binned into 20 base-pair bins, where each bin is represented by the bin-center. Y-axis shows the normalized frequency distribution of the MED-1 element.

**Figure 14:**
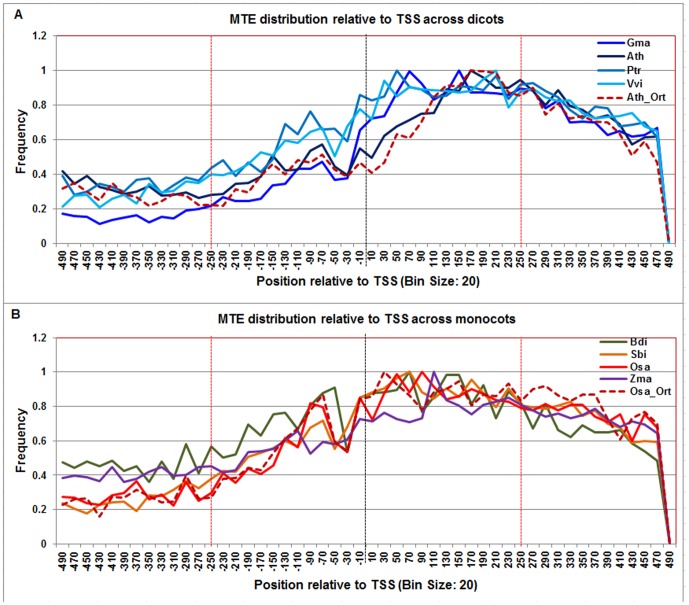
Normalized frequency distribution profile of MTE with respect to TSS across monocots and dicots. Panel A: Genome-wide positional distribution of MTE frequency profile across four dicots: *Arabidopsis thaliana* (Ath - solid navy blue), *Glycine max* (Gma-solid dark blue), *Populus trichocarpa* (Ptr –solid blue sapphire), and *Vitis vinifera* (Vvi -solid blue green). Genome-wide functional distribution profile of MTE based on the ortholog mapping of Ath with rest of the dicots (Ath:Gma, Ath:Ptr, and Ath:Vvi) is shown in dotted brown colored line. Panel B: Genome-wide positional distribution of MTE element frequency profile across four monocots: *Brachypodium distachyon* (Bdi-solid bronze yellow), *Oryza sativa ssp. japonica* (Osa-solid red), *Sorghum bicolor* (Sbi-solid bronze), and *Zea mays* (Zma -solid purple). Genome-wide functional distribution profile of MTE based on the ortholog mapping of Osa with rest of the monocots (Osa:Bdi, Osa:Sbi, and Osa:Zma) is shown in dotted brown colored line. X-axis shows [−500,+500 with respect to TSS] that is binned into 20 base-pair bins, where each bin is represented by the bin-center. Y-axis shows the normalized frequency distribution of the MTE element.

**Figure 15:**
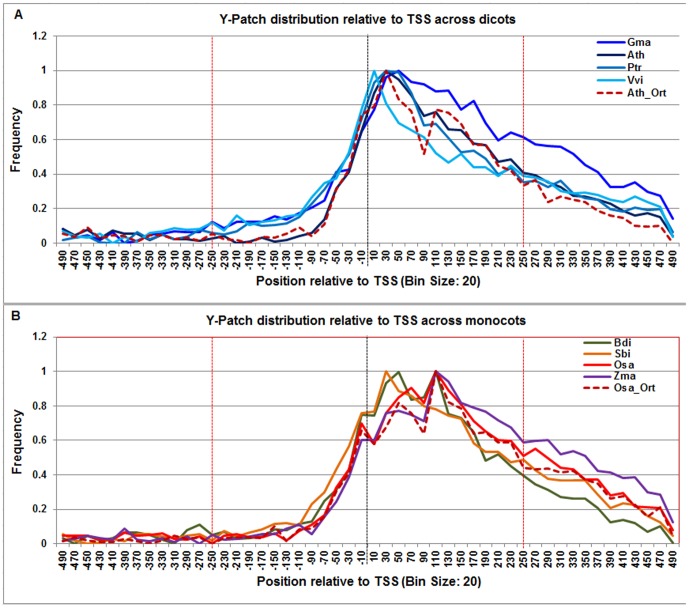
Normalized frequency distribution profile of Y-patch with respect to TSS across monocots and dicots. Panel A: Genome-wide positional distribution of Y-patch element frequency profile across four dicots: *Arabidopsis thaliana* (Ath - solid navy blue), *Glycine max* (Gma-solid dark blue), *Populus trichocarpa* (Ptr –solid blue sapphire), and *Vitis vinifera* (Vvi -solid blue green). Genome-wide functional distribution profile of Y-patch based on the ortholog mapping of Ath with rest of the dicots (Ath:Gma, Ath:Ptr, and Ath:Vvi) is shown in dotted brown colored line. Panel B: Genome-wide positional distribution of Y-patch element frequency profile across four monocots: *Brachypodium distachyon* (Bdi-solid bronze yellow), *Oryza sativa ssp. japonica* (Osa-solid red), *Sorghum bicolor* (Sbi-solid bronze), and *Zea mays* (Zma -solid purple). Genome-wide functional distribution profile of Y-patch based on the ortholog mapping of Osa with rest of the monocots (Osa:Bdi, Osa:Sbi, and Osa:Zma) is shown in dotted brown colored line. X-axis shows [−500,+500 with respect to TSS] that is binned into 20 base-pair bins, where each bin is represented by the bin-center. Y-axis shows the normalized frequency distribution of the Y-patch element.

The genome-wide distributions of the above-mentioned CPEs were predicted in known protein coding genes' core promoter sequences flanked with 5′UTR in eight plant genomes using PWM (Figures 3–15). For each genome, the frequency distribution profile of the individual CPE was constructed and compared against its profile generated from the randomly generated background sequences to locate CPE abundance signal (background profiles are not shown). Only those promoter regions where the foreground signal was statistically significant from the background signal were considered as the candidate CPE localization ranges (see methods for details). The frequency distribution profiles of these CPEs in *A. thaliana* and *O. sativa* ssp. *japonica* were selected as representative models to compare dicots and monocots, as shown in Figures S3 and S4 respectively. The similarities and differences in promoter architecture between monocots and dicots, underscored by differences in DNA free energy profile studies, provided insights into the positional preference of the CPEs and reduced the false positive predictions (Table S4).

Furthermore, we also predicted these CPEs based on the gene conservation across genomes by selecting Ath (orthologous pairs of Ath:Gma, Ath:Ptr, and Ath:Vvi) and Osa (orthologous pairs of Osa: Bdi, Osa:Sbi, and Osa:Zma) ortholog gene promoter sequences. The brown dotted line in Figures 3–15 corresponds to the orthology-based CPE frequency distribution profiles (see methods for details). The predicted range of each CPE for each monocot and dicot based on positional and orthologous gene conservation is given in Table S4. For each CPE motif, a consensus localization range was determined independently in the monocot and dicot groups using both qualitative and quantitative measures. To see the similarities and differences in promoter sequences across monocots and dicots, the comparison of each motif across monocots and dicots is described next.

The **Inr** motif signal spanned over a wider range in dicots as compared to the monocots. In dicots, the first significant Inr signal peak appeared at −20 and continued till +240 while in monocots it started at −60 and stretched till +60, showing group level differences in the general genome wide organization of the Inr signal between dicots and monocots (Figure 3). In monocots, a sharper peak further appeared downstream of TSS from +100 to +120. Therefore, monocots had a relatively focused TSS motif signal as compared to dicots. The Inr profile based on Arabidopsis orthologs and rice orthologs also agrees with respective dicot and monocot Inr profiles (Table S4).

The **TATA-box** binding site was remarkably conserved across all dicots and monocots and in ortholog sequences, with a sharp peak ranging from −60 to −20 except Vvi and Sbi that ranged from −70 to −20. The difference in relative TATA abundance peaks among species is due to the difference in total number of genes annotated with 5′UTR in the corresponding species (Figure 4).

The **CCAAT-box** detection signal, though found to be present upstream of the TSS across all plant genomes, ranged from −120 to −40 in dicots and from −460 to −140 in monocots, a pattern that was recapitulated in the profiles built for Arabidopsis and rice ortholog sets respectively (Figure 5).

The **BREu** motif signal appeared bi-modal in dicots and uni-modal in monocots. In both taxa, the BREu signal trended upward from the TATA-box and peaked ∼ 110 bp beyond the TSS. However, the TATA-box was found to distinctly segregate the first signal peak (ranging from −100 to −40) from the second signal peak (ranging from +40 to +200) in dicots (Figure 6). Notably, in monocots, the BREu signal was predominantly downstream of TSS with a broad peak that declined gradually beyond +180 (Figure 6). The BREu frequency distribution in the Arabidopsis orthologs and rice orthologs respectively agreed with dicots and monocots frequency distribution profiles.

The **BREd** frequency distribution appears bimodal in both dicots and monocots (Figure 7). In dicots, small distance separated the two peaks which ranged from −80 to −40 and from −20 to +10 (Figure 7). As observed for BREu, the region separating these BREd peaks coincides with the TATA-box peak. However, in monocots, the two peaks were more distantly separated, the first ranging from −140 to −100 and the second from −40 to +40. This suggested that elements in addition to the TATA-box may be intervening. In our analysis, the monocot Inr and DCE-S1 are the only CPEs that overlap this region.

The **GC-box** signal exhibited a broad range (−70 to +250) in both dicots and monocots (Figure 8). Although a similar range was found in rice orthologs, the GC-box distribution was narrower in the Arabidopsis ortholog set. The PWM given in JASPAR database [102] for GC-box was derived from 502 unrelated promoter sequences from four eukaryotic RNA polymerase II promoter elements [108]. Given the broad range of GC-box signal in both monocots and dicots, it could be inferred that GC-box might be delocalized in plants.

The **DCE-S1** frequency distribution showed a peak at +100 to +160 in all eight plant genomes (Figure 9). In monocots, an additional distinct peak was also evident from −40 to +40, which substantially overlaps with Inr and BREd signals around TSS and partially overlaps with the Y-patch signal (see below). The respective ranges in dicots and monocots were also confirmed by the Arabidopsis orthologs and rice orthologs.

The **DCE-S2** signal is multimodal in dicots and monocots (ranging from −140 to −100, −20 to +1, and +100 to +160) (Figure 10). The DCE-S2 peak in monocots around the TSS is distinctive. These multi-modalities in DCE-S1 and DCE-S2 motif signals could suggest a diverse role of DCE-variants in Pol-II PIC formation, depending on the position of these motifs around the TSS. The DCE-S2 profile in rice orthologs also confirmed the observed range in monocots; however Arabidopsis orthologs showed a peak from +280 to +300 in addition to the observed range (−20 to +1 and +100 to +210).

The **DCE-S3** foreground signal was not statistically different from background signal and therefore, the prediction of DCE-S3 was not included in this study.

The **DPE** motif signal covered a broad range both in dicots (+40 to +360) and monocots (+100 to +400) (Figure 11). Given this long and flat profile, it can be hypothesized that the consensus sequence of DPE used to develop the PWM, lacks sufficient specificity to refine the range of DPE elements in plants. The DPE profile in Arabidopsis and rice orthologs also confirmed this range.

The **XCPE-1** signal differs between dicots and monocots. In dicots, it showed peaks at −70 and drops at −30 and sharply plateaued from around +100 till +180, gradually falling off thereafter (Figure 12) whereas in monocots, XCPE-1 signal is shifted downstream with an initial rise at +60 and reaching a broad plateau from +130 to +240. The profiles based on the Arabidopsis orthologs and rice orthologs also agreed with these results.

The **MED-1** showed a robust and consistent frequency distribution across all plant genomes. It appeared to be bimodal, with a peak around −10 and other signal from +100 to +200 and the profiles based on Arabidopsis and rice orthologs also confirmed this range (Figure 13, Table S4).

The **MTE** frequency distribution profile was found to be consistently high across all plant genomes between +20 to +220. However in monocots, it starts at −20 and ends at +240 whereas in dicots it starts at +20 and ends at +220. This observation was also confirmed in respective model monocot and dicot orthologs (Figure 14).

Our predictions showed a very strong and robust **Y-patch** frequency distribution across all plant genomes between +20 to +80. However, the overall signal span was shorter in dicots (from −20 to +80) and longer in monocots (from +20 to +160) (Figure 15). The Y-patch profile in the Arabidopsis orthologs also agreed with the observed dicot range.

To assess the reliability of our prediction methodology, we applied it to a set of ∼2000 experimentally determined *D. melanogaster* promoters available from the Eukaryotic Promoter Database (EPD) [109] using PWMs from the JASPAR POLII database [102]. Out of the 13 core promoter elements examined above, four CPEs (Inr (+1), TATA-box (−25), DPE (28–33 bps), and MTE (17–22 bps)) have been experimentally confirmed in Drosophila promoters [22], [35], [107]. We compared our prediction results for these four CPEs with their positional distribution described in the literature. Inr element was found to be present from −20 bp upstream of TSS to +19 bp downstream of TSS, TATA-box from −40 to −21 bp upstream of TSS, MTE from +1 to +19 bp downstream of TSS, and DPE from +20 to +39 bp (Figure S5). These results are in close proximity to the reported literature on these CPEs [22], [35], [107].

### Genome-wide prevalence of TATA-containing, TATA-less, and CPE-less promoters

Based on the putative range of each CPE (Table S4), we calculated the percentage distribution of each CPE for eight genomes (Table S5). There is statistically significant difference in the prevalence of each CPE (except TATA-box, DPE and Y-patch) between Arabidopsis and rice (two sample t-test = 0.009) (Table S5). On the average, Inr and DPE are significantly prevalent in dicots whereas CCAAT-box, GC-box, XCPE1, MED1 are significantly prevalent in monocots (Table S5).

We categorized promoters into three broad classes: 1) promoters having a putative TATA-box (TATA(+)), 2) promoters lacking a putative TATA-box, but containing at least one other putative CPE (TATA(−)), and 3) promoters lacking all of the thirteen CPEs (CPE(−)). An account of the TATA(+), TATA(−), and CPE(−) genes for each genome is given in Table 1. Dicots and monocots have ∼18% TATA(+) promoters, except Zma and Sbi in which ∼13% and ∼22% of their respective promoters contained TATA-box (Table 1). On average, ∼81% of dicot and monocot promoters were categorized as TATA(−). Further, we noted that on average 1.45% dicot and 0.76% monocot promoters lacked known CPEs within the selected range of the putative promoter sequences. Given the low proportion of transcripts with annotated 5′UTR, especially Bdi and Sbi in which fewer than 50% transcripts are annotated with 5′UTR information, these numbers may change in future as the genome annotations improve (Table 1).

**Table 1:** 5′UTR annotated transcripts in TATA(+), TATA(−) and CPE(−) class of promoters.

	TATA(+)		TATA(−)		CPE(−)		Genome	
Species	Transcripts	%	Transcripts	%	Transcripts	%	Transcripts with 5′UTR	Annotated 5′UTR (%)
*Arabidopsis thaliana* (Ath)	4907	18.1	21638	79.9	551	2.0	27096	77
*Glycine max* (Gma)	7196	19.3	29453	79.1	593	1.6	37242	67
*Populus trichocarpa* (Ptr)	4167	16.4	20904	82.4	293	1.2	25364	59
*Vitis vinifera* (Vvi)	2873	16.8	14038	82.2	174	1.0	17085	57
*Brachypodium distachyon* (Bdi)	2000	17.4	9407	81.9	78	0.7	11485	37
*Oryza sativa* (Osa)*	3614	18.2	16210	81.5	75	0.4	19899	65
*Sorghum bicolor* (Sbi)	2871	22.3	9993	77.5	36	0.3	12900	36
*Zea mays* (Zma)*	3750	13.0	24536	85.3	488	1.7	28774	73
Dicot average		17.67		80.88		1.45		
Monocot average		17.72		81.53		0.76		
Dicot standard deviation		1.32		1.66		0.46		
Monocot standard deviation		3.78		3.20		0.65		

* Osa and Zma – based on filtered gene set (after removing transposons).

### Classification of the promoters based on combinatorial modules

To understand which and how many CPE combinations were more frequent within the group of dicots and monocots, we further computed the combinatorial grouping of the CPEs, also known as combinatorial modules (see methods). There were 1,800 unique combinatorial modules that were common across four dicots that covered around 81% of the dicot promoters (Table S6). Likewise, there were 1,323 unique common combinatorial modules across four monocot genomes (Table S7) that covered about 70% of the monocot promoters. We analyzed the top 221 dicot and 216 monocot modules accounting for 51% promoters in each group. Y-patch and GC-box were found to be prevalent across all the dicot and monocot genomes. The combinatorial module of Y-patch, GC-box, XCPE1, and MTE dominated monocot promoters whereas the combinatorial module comprised of Y-patch, Inr, DPE, and GC-box was more prevalent in dicot promoters. Across all dicots and monocots, DCEs were found more frequently with Y-patch and/or BRE and less frequently with Inr suggesting diverse role of DCE in PolII PIC formation. A complete overview of the most frequent unique combination of CPEs modules is given in Tables S6 and S7.

To further determine the prevalence of CPEs for each combinatorial module, the dataset was partitioned with respect to the presence and absence of the TATA-box (Table S8). The prevalence of each CPE in the presence and absence of TATA-box was not statistically different when compared within dicots or monocots (Table S8). However, when comparing Arabidopsis and rice, we detected significantly different combinatorial modules (Table S8). Among TATA(−) modules, the GC-box, CCAAT-box, DCE and MED-1 elements showed higher prevalence in rice whereas in Arabidopsis, the DPE and Inr were more prevalent (Table S8). Similar results were found when partitioning was done on the basis of presence and absence of Inr, DPE or Y-patch, reflecting underlying differences between monocots and dicots with respect to CPE distribution (data not shown).

### Functional enrichment analysis of TATA-containing, TATA-less and CPE-less genes using gene ontology

To understand possible relationships between promoter structure and gene function, we tested for Gene Ontology (GO) enrichment among the TATA(+), TATA(−), and CPE(−) genes in Arabidopsis. Tables S9 and S10 show results of this analysis for the molecular function and biological process categories of the gene ontology respectively. In general, little overlap was observed between enriched GO categories amongst the three classes of promoter.

**Figure 16:**
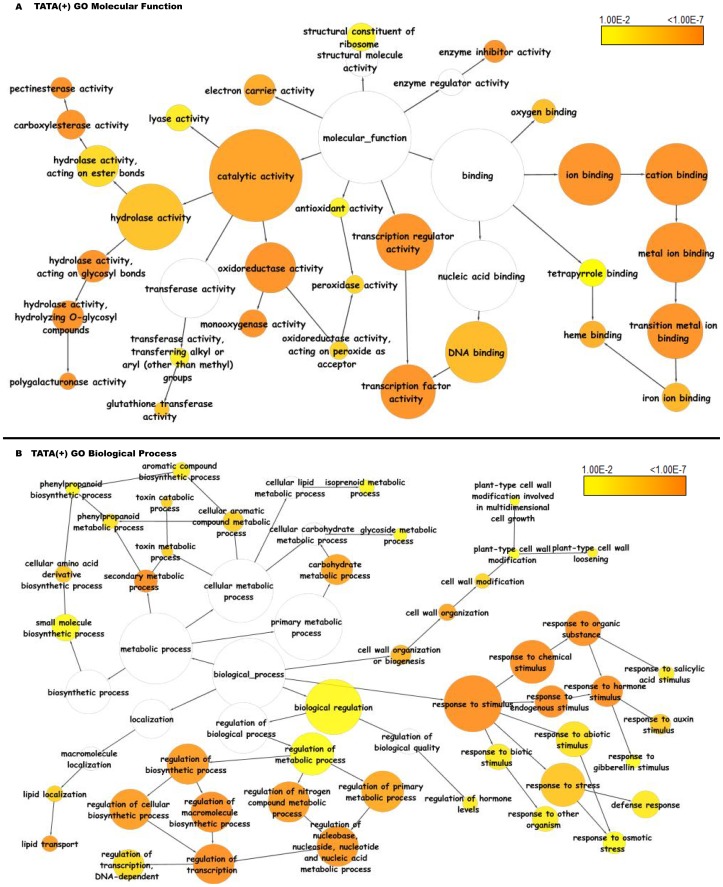
Functional annotation based on gene ontology molecular functions and biological processes of TATA containing genes in *Arabidopsis thaliana*. Significantly overrepresented GO terms based on GO molecular functions and biological processes were visualized in Cytoscape. The size of a node is proportional to the number of genes in the GO category. The color represents enrichment significance - the deeper the color on a color scale, the higher the enrichment significance. White color nodes are not enriched but show the hierarchical relationship among the enriched ontology branches.

The TATA(+) class showed significant overrepresentation of molecular functions involved in transcription regulation, ion and DNA binding activities, electron transport and enzyme inhibitor activities (Figure 16A). The biological process enrichment showed categories that included response to stress, abiotic, biotic and hormonal stimuli, regulation of carbohydrate and nucleic acid metabolic process, secondary metabolic process, lipid transport and cell wall modification (Figure 16B).

**Figure 17:**
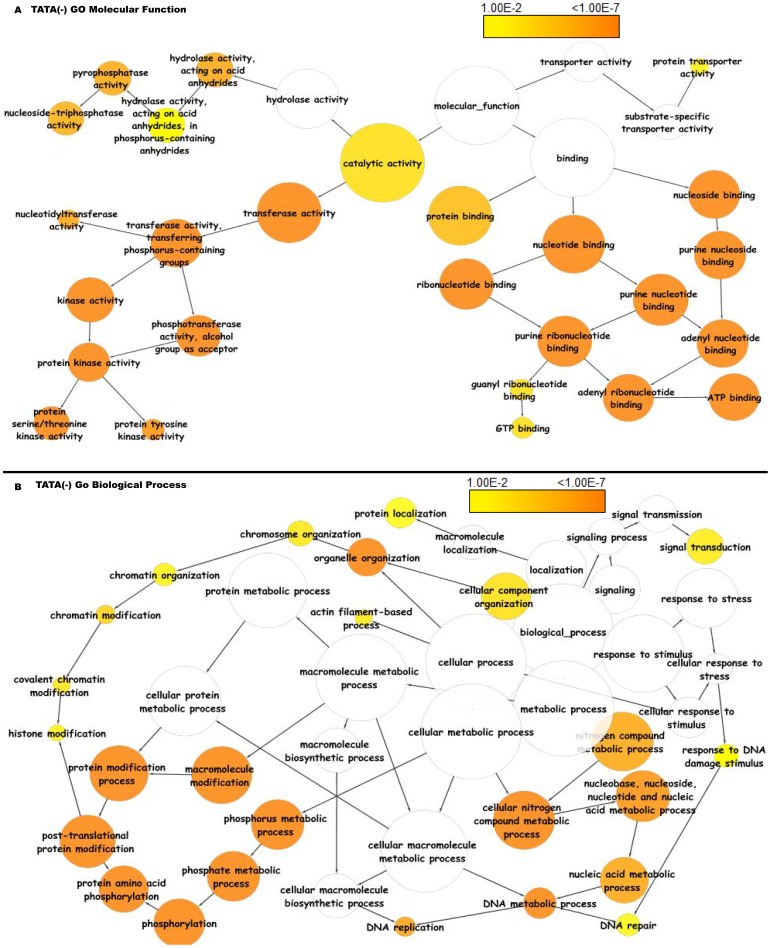
Functional annotation based on gene ontology molecular functions and biological processes of TATAless genes in *Arabidopsis thaliana*. Significantly overrepresented GO terms based on GO molecular functions and biological processes were visualized in Cytoscape. The size of a node is proportional to the number of genes in the GO category. The color represents enrichment significance - the deeper the color on a color scale, the higher the enrichment significance. White color nodes are not enriched but show the hierarchical relationship among the enriched ontology branches.

The TATA(−) class showed enrichment in a variety of molecular functions, including transferase activities, hydrolase activities, and various nucleotide related binding activities (Figure 17A). These genes were enriched in biological processes related to nitrogen and phosphorous metabolism (Figure 17B).

**Figure 18:**
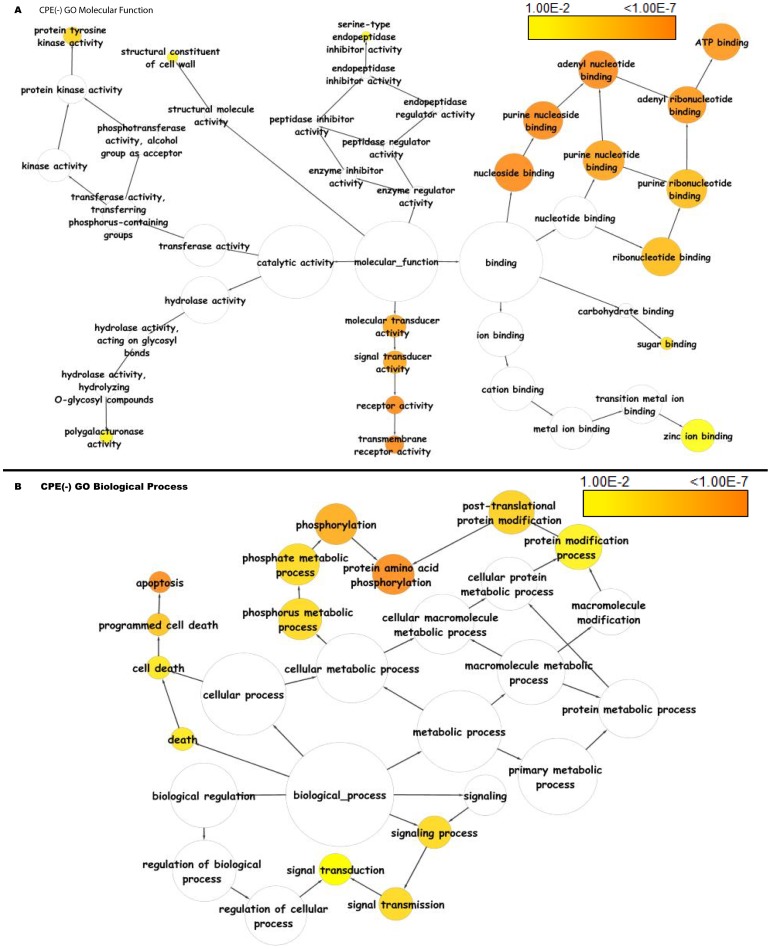
Functional annotation based on gene ontology molecular functions and biological processes of coreless genes in *Arabidopsis thaliana*. Significantly overrepresented GO terms based on GO molecular functions and biological processes were visualized in Cytoscape. The size of a node is proportional to the number of genes in the GO category. The color represents enrichment significance - the deeper the color on a color scale, the higher the enrichment significance. White color nodes are not enriched but show the hierarchical relationship among the enriched ontology branches.

The CPE(−) class showed evidence of unique enrichment in genes involved in ATP binding, signal transduction activities, apoptosis as compared to the TATA(+) and TATA(−) class of genes (Figures 18A,B).

## Discussion

The genome-wide characterization of the gene expression regulation is a complex process and presents one of the major challenges in comprehensive identification of the transcriptional regulatory elements in plant genomes. The RNA polymerase II core promoter, also known as gateway to transcription [110], is a complex regulatory element that provides considerable diversity to the core promoter structure and function [111]. Several CPEs have been previously identified in eukaryotes [31], [49], [59], [112], [113], [114], [115], [116], [117], [118], however, they have not been well studied in evolutionarily diverse plants. In addition, there are promoters that lack any known CPEs suggesting that one or more novel classes of motifs might be involved in the transcription regulation. Here, we have used a systematic and unbiased high-throughput computational approach that involves sequence and structural properties of DNA to identify the core promoter region and CPEs across monocots and dicots. Our results suggest that many CPEs identified in animals are evolutionarily conserved in plants, thus indicating their essential role in transcriptional regulation. Since, majority of the CPEs were derived from animal genomes, it is possible that their positional conservation profiles might be slightly off and broader than expected in plants. However, we speculate that with the knowledge of the plant specific PWMs, CPE signal profile can be expected to improve. We have redefined basic promoter features and analyzed the conservation and diversity of plant promoters on a genome-wide scale.

The conserved motifs can be detected by analyzing distribution profiles in a large set of promoter sequences and promoter architecture can thus be deduced. The genome-wide prevalence of these CPEs across four monocots and four dicots was based on the positional conservation of the regulatory elements that share common content features. By using positional conservation, false positive signals can be reduced significantly and therefore, biologically relevant motifs can be discriminated from the false predictions [94], [95], [96]. Furthermore, it has been reported that the orthologous genes have also been used to identify the regulatory modules that are conserved between species belonging to different plant families [119], [120]. Conserved DNA motifs show that the preferential appearance of a set of sequences might be due to evolutionary pressure and thus suggest the potential functional role in transcription regulation or some other biological processes. Therefore, based on comparative genomics studies, the CPEs that are commonly conserved among orthologous genes across monocots and dicots are more likely to be functional. Generally, the transcription factor binding sites of core promoters showed conservation between monocots and dicots, with differences that suggest distinct promoter architectures possibly due to evolutionary divergence between these groups. Randomly selected real genomic sequences were used for background motif signal, which further helped in understanding the differences between promoter and non-promoter regions. Our results demonstrate that motif signal localization and positional-conservation can greatly improve the identification of functional CPEs in monocots and dicots. These putative functional core promoter elements can be experimentally confirmed through experimental approaches like yeast-1-hybrid (Y1H) [4] and chromatin-immunoprecipitation (ChIP) assays [5].

We believe that factors such as CPE length, and the quality and consistency of genome annotation methods in TSS identification play a vital role in determining an optimal bin-size. While genome annotation efforts frequently include methods to pin-point TSS location to an exact base for each gene, these efforts are frequently limited by lack of transcriptional evidence, or complicated by phenomena such as alternate splicing or multiple start sites. Promoters are also known to exhibit heterogeneity, with some exhibiting a sharp window within which transcription may start while transcription in others occurs over a dispersed range [121], [122]. These phenomena further complicate annotation efforts, which generally attempt to define a single TSS than a range of start sites. In the end some genes will have more reliable annotation than others. Methods to characterize DNA physical properties and core promoter motifs, as described here, may hold promise to augment and refine annotation methodologies in the future.

### DNA free energy profiles of core promoter region

Our study of DNA free energy profiles in both dicots and monocots demonstrated consistent distinction between promoter and non-promoter regions on the basis of DNA physical properties. Our observations are also consistent with a number of previous studies on DNA energy profiles [81], [123], [124], [125], [126]. While the non-regulatory genome's energy profile was consistently flat, the regulatory genome's profiles exhibited interesting properties. Both dicots and monocots showed similar average free energy profiles, characterized by a sharp peak of instability near the TSS, which punctuated an overall trend of increasing stability ranging across the promoter and downstream regions. Differences in baseline free-energy profiles across different species are associated with differences in their GC content. Monocots have higher GC content (50–65%) as compared to dicots (35–45%). Nevertheless, despite the impact of these differences on absolute free-energy, the shape of the curves (i.e. the pattern of relative changes in free-energy across promoter space) showed remarkable conservation across species. The most dynamic portion of the free-energy profile falls within a 150 bp window centered on the TSS, wherein free-energy can vary by up to 3.5 kcal/mol (Figure 2). At least two local peaks could be discerned, the larger at ∼ −30 and the smaller at the TSS itself. Our observations provide basis for the potential future use of free-energy profiling as an *in silico* annotation tool to predict the locations of core promoters and sites of transcription at a genomic scale. DNA free energy profiles or stability profiles (based on the melting of DNA double strand) provide insights into the physicochemical properties of the promoter region [127], [128] and can be helpful in gaining understanding of nucleosome organization and chromatin structure.

### Positional and orthologous conservation of CPEs across eight genomes

The PWMs of TATA-box, Inr, and CCAAT-box were constructed from plant sequences in PlantProm database [101]. Similarly Y-patch PWM is derived from rice genome [15]. All other PWMs were derived from Drosophila and other animals or fungi and were taken from JASPAR POLII database [102]. We analyzed the distribution between TATA-box and Y-patch promoters with 5′ UTR length, and found that about 80% and 51% of the TATA-box containing promoters have 160 bp or smaller 5′UTR length whereas 77% and 61% of the Y-patch containing promoters have 210 bp or smaller 5′UTR length in *A. thaliana* and *O. sativa japonica* respectively.

Based on the genome-wide percentage of each CPE in eight genomes, we found that TATA-box was present in around 16–22% of the promoters. In Arabidopsis and rice, it was present in around 18% of the promoters. It is consistent with the recent publications on TATA-box in Arabidopsis and rice [15], [129], [130]. However, earlier work in Arabidopsis by Molina and Grotewold [14] reported around 29% of promoters that contain TATA-box in a set of highly expressed genes (around 12,749 transcripts). The higher percentage of TATA-box in their studies can be due to ascertainment bias of highly expressed genes and therefore, is likely to be overrepresented in the smaller gene sets.

Additionally, we found that the positional preferences of the BREu and BREd motifs in plants are different from animals, as the latter were reported to have these motifs immediately upstream and downstream of the TATA-box respectively [19], [128]. The BREu motif signal appeared to be bimodal in dicots surrounding TATA-box, but the second peak of BREu extends far downstream whereas in monocots, BREu is unimodal but it is downstream of TSS. BREd abundance signal appears to be bimodal in both dicots and monocots. Based on these results and given the differences in location, BRE may be expected to function differently in plants compared to animals and found to be less associated with TATA-box. Our results also differ from a previous report that BRE motifs are missing in plants, as well as yeast [131]. Other CPEs also showed differences in abundance signal in dicots as compared to monocots. The GC-box signal appeared more robust in monocots than dicots, as did DCE-S1 and DCE-S2. We observed that the predicted range of plant CPEs on the promoter is broader than reported in literature [2], [14], [98], possibly because of the PWMs derived from animal genomes. Furthermore, previous studies in the plants used a relatively narrow core window size of TSS±50 [14], [77] and thus precluded the examination of distal downstream elements like DCE, DPE, MTE, and MED that our study explores. This is expected as the distal elements usually cluster after +100 base pairs. We observed that the CPE abundance signal of potentially functional elements are consistently better in monocots than in dicots, possibly due to differences in the quality of genome annotations and the relative degree of evolutionary distance among closely related species. It should be noted that all species in our monocot group are in the grass family, thus representing less diversity than the species in the dicot group.

### Classification of promoters based on combinatorial modules

The role of CPEs is more diverse than previously thought and therefore, combinatorial modules of CPEs present in the promoter region can act to regulate specific classes of genes as well. Based on our *in silico* analysis of the core promoters across eight plant genomes, we classified plant promoters into three types, TATA(+), TATA(−), and CPE(−), and sought to understand how these classes relate to combinatorial modules of regulatory domains. It has been reported that in mammalian promoters in mouse and human, TATA(+) promoter is associated with sharp TSS clusters, whereas, the TATA(−) promoters have broad type TSS clusters [132], [133]. Based on our genome-wide studies, plant promoters appear to have dispersed promoter and thus can not be associated with sharp or broad TSS clusters. However, TATA-box appeared to be the best recognized core element based on its signal, which is highly conserved across plant genomes.

In the TATA(−) class of promoters, the presence of Inr, DPE, and MTE, which have also been found in a subset of TATA(+), can provide additional insights into the transcription of TATA(−) genes. Though the sequences of Inr and DPE elements are different than TATA-box, TFIID can recognize and bind directly to these CPEs by using the TAF subunits [31], [134]. Based on *in silico* and experimental studies from literature related to RNA Pol-II dependent transcription initiation, TFIID, though not a universal factor, seems to be a key driver of the RNA Pol-II PIC assembly for TATA-containing core promoters as well as for other core promoters primarily driven by core elements like DPE, MTE, Inr, and BRE [19], [22]. Interestingly, our studies suggested that the BREs (BREu and BREd) show an independent distribution among core promoters. Based on the prevalence of core promoter elements in combinatorial modules among dicots and monocots, GC-box and Y-patch were found to be the most prevalent CPEs that participated in putative combinatorial modules either individually or jointly with one or more of the rest of the CPEs across all the dicot and monocot genomes. In addition to this, XCPE1 and MTE dominated monocot promoters whereas Inr and DPE dominated dicot promoters. We found similar trend in the prevalence of CPE in the promoter sequences that do not contain TATA-box. It seems, in absence of TATA-box, Inr and DPE play significant roles in dicots whereas GC-box, CCAAT-box, DCE and MED-1 elements play regulatory role in monocots. The DPE motif was reported in the literature to be functionally dependent on the Inr in *D.melanogaster* [21]. However, in our studies, DPE showed more prevalence in the promoter sequences of dicots that did not contain Inr element. Similarly, Inr had higher prevalence in the promoter sequences of dicots that did not contain DPE. We further analyzed predicted combinatorial modules to find out if combinatorial constraints (INR/DPE, INR/MTE, and INR/DPE/MTE) could help refine the respectively predicted signals of DPE and MTE motifs. Contrary to the speculation, further analysis of these CPEs as combinatorial modules did not narrow down the DPE and/or MTE signal. It can be due to broad positional range of the Inr and the percent distribution of both MTE and DPE is relatively higher in the Inr(−) class of promoters than the Inr(+) class of promoter.

### Functional enrichment of TATA(+),TATA(−) and CPE(−)

There are various reports on heterogeneity in the plant core promoter types that vary with respect to CPE composition [30], [129], [135], [136], [137]. The core promoter type also seems to correlate with gene structure and expression characteristics. For example, in spite of the absence of core elements, the CPE(−) type promoters manifested constitutive gene expression, whereas TATA(+) promoters were found in genes with tissue-specific gene expression [130]. Functional annotation of CPE based on GO analysis in yeast, human and Arabidopsis had reported that the TATA-type promoters were enriched in environmental response genes whereas TATA-less genes are more often involved in housekeeping processes [13], [16], [17], [18], [129]. Our studies on GO enrichment analysis also suggested that the TATA(+) class of promoters were mainly involved in stress responses and TATA(−) were involved in housekeeping functions, while CPE(−) were mainly involved in signaling activities.

All of the mechanisms involved in the expression and regulation of genes ultimately depend on the core promoter. It was found that nearly all of the Homeotic (*Hox*) genes that lack TATA-box in *D. melanogaster* have DPE-dependent promoters [121]. It is well reported that the *Hox* genes code for transcription factors which are necessary for the sequential development of many anatomical structures, and the expression and regulation of these genes are dependent on the CPEs present in their promoters [121]. Based on our GO analysis of DPE containing promoters that lack TATA-box in Arabidopsis and rice, we also found that these genes were mainly enriched in developmental process, especially during shoot system development, flower development and proximal/distal pattern formation. This example underscores how evolution has used the diversity of CPEs to regulate the expression of an important class of proteins.

### Conclusion

In this paper, we have presented a strategy for annotating CPEs by computational prediction at the whole genome level in different plant genomes. Identification and characterization of the core promoter binding site motifs of the transcription factors participating in the formation of PIC complex will help us understand the core promoter architecture and establish the processes by which plant basal transcription machinery functions. Our analysis of CPEs across all eight genomes revealed that the predicted range of most of CPEs in our study is broader than typically reported in the literature. This may be due to inherent differences in core promoter architecture in plants as compared to *E.coli*, yeast, Drosophila and mammalian genomes [1], [16], [21], [22], [23], [30], [31], [98], [131], [138]. However, there can be few other possible reasons: (i) if the majority of promoters are of the dispersed type as opposed to the focused type, corresponding core DNA sequence motifs are likely to be spread over a broader range as well [110], [139]; (ii) for a study of this size, we made a conscious decision to report our finding at the genome-group level and highlight the monocot and dicot specific differences where necessary without compromising comprehension; (iii) not all genomes in a group have the same quality of annotation, for example, *Arabidopsis thaliana* by far is the best annotated genome among dicots, while rice annotation likely exceeds other genomes among monocots; (iv) not all CPE consensus sequence and PWMs are as robust as TATA-box and Y-patch across the board [75]; (v) though the window size of 20 bp was found to be optimal for genome-wide CPE profiles across species, this might have an effect on the broader CPE distribution range in our study; and (vi) most of the PWMs used in this study were developed for non-plant species and may have reduced specificity when used in plants. As the genome annotations improve over time and the 5′UTR information becomes available for rest of the genes, the CPE distribution profiles discussed in this study might need to be reassessed using our recommended methodology. Nevertheless, this study demonstrates that our prediction methodology is reliable, robust, tunable, and automatable. Due to the large number of reference genomes currently available, we were able to study core promoter element localization and their positional-conservation across species. Thus our comprehensive analysis of plant promoter sequences can be exploited in developing a full-fledged *in silico* tool for plant promoter prediction. Based on these studies, we believe that results are sensitive and specific enough to guide verification by subsequent wet lab experiments.

There is very little knowledge of core promoter elements in plants and our study offers a new insight in the field with an important distinction of dicots vs. monocots. Our study documents, in a single comprehensive catalog, the computational prediction and prevalence of thirteen known core promoter elements in four monocots and four dicots. The core promoter region free energy profile seems to possess a characteristic signature that distinctly differentiates it from the non-promoter genomic regions and has the potential to be used for delineating the promoter region as well as for computational TSS identification. It can help build better computational models for predicting the TSS in the promoter region, which remains one of the most challenging problems. We also examined the prevalence of each CPE in the combinatorial modules in dicots and monocots and in the presence and absence of TATA-box in all eight genomes. TATA-box was found to be present in 16–22% of the plant promoters. GC-box, XCPE1 and Y-patch were found to be the most prevalent CPEs across all eight genomes. In conclusion, this study expands the CPE repertoire in plants, providing impetus for future wet-lab research.

## Materials and Methods

### Genome Sequence

The core promoter sequences [TSS±500] of protein coding genes with known 5′UTR information were extracted from the Gramene core databases (version 34b) [www.gramene.org] for four monocots (Bdi, Osa, Sbi, Zma) and four dicots (Ath, Gma, Vvi, Ptr). To see cross-species conservation of CPEs based on orthologs, *A.thaliana* and *O.sativa ssp japonica* orthologous protein coding genes with sequence identity ≥50% with their respective dicots (Ath:Gma, Ath:Ptr, Ath:Vvi) and monocots (Osa:Bdi, Osa:Sbi, Osa:Zma) were selected for orthologous conservation study. 9225 and 7958 orthologous protein coding genes for Ath and Osa were selected for this study. The ortholog dataset was retrieved from Gramene biomart [140] that itself depends on the Ensembl Compara pipeline, which is based on a phylogenetic analysis[141].

The promoter sequences for *Drosophila melanogaster* were extracted from Eukaryotic promoter database [http://epd.vital-it.ch/] [109] as a gold standard to test the reliability of our CPE predictions.

### DNA free energy profiles of core promoter region

DNA free energy profiles of the promoter regions and randomly generated background sequences as negative control related to eight plant genomes were constructed using PromPredict – an algorithm based on GC content of the genome as well as difference in the GC content of the promoter region and the genomic sequence flanking the promoter region [103]. It uses experimentally established dinucleotide free energy values in a sliding window of size 15bps as proposed by Allawai and Santalucia [142] and Santalucia [143]. An average free energy profile was calculated by taking the mean value of free energy at each position over all the promoter sequences from a genome.

### Positional and orthologous conservation of CPEs across eight genomes

For positional conservation, we analyzed the promoters for which 5′UTR information was available for all eight genomes. For orthologous conservation, *Arabidopsis thaliana* as a dicot model and *Oryza sativa ssp. japonica* as a monocot model were selected as mentioned before. It provided an additional approach to increase the confidence in putative CPEs predictions made based on the positional conservation.

Search Tool for Occurrences of Regulatory Motifs (STORM) was used to identify motifs based on predefined PWMs [60], [104], [105]. STORM has been successfully used in whole genome mapping and analysis of active promoters in mouse embryonic stem cells and adult organs using known vertebrate motif PWMs from TRANSFAC [144] and JASPAR [60],[102]. For a given genome, the promoter sequences were processed through STORM to detect the presence of CPE binding site consensus sequences. STORM does not make any prior assumption about distribution of nucleotides in the promoter sequences rather it uses actual nucleotide composition of the genome to increase accuracy in estimating statistical significance of the binding site predictions. We considered only those putative cis-regulatory motifs that were overrepresented in promoter sequences of protein coding genes as compared to a background set of same number of random genomic sequences. STORM runs for CPE predictions were set to detect each core-element at a p-value ≤0.001. STORM used this p-value to calculate core-element specific scoring threshold that depends on the information content and length of each motif [145].

We generated and tested several background models specifically derived from corresponding real genomic sequences from non-regulatory regions as well as a pseudo random background model designed from a large set of sequences that had been generated artificially *in silico* according to the real nucleotide composition of the genomes in our data set. It established motif's positional signal detection base-line threshold. The range in which foreground CPE abundance was at least 1.5 standard deviations higher than the background CPE abundance was considered as a candidate binding site region. Further, overlaying of individual species frequency distribution profiles within monocots and dicots provided positional conservation of the binding site region across species. Overlaying of frequency distribution profiles based on *A. thaliana* orthologous gene promoters among dicots and *O. sativa japonica* orthologous gene promoters among monocots provided further confidence in cross species conservation of CPEs. Based on these approaches, the consensus range for each CPE was determined independently in the monocots and dicots.

### Classification of promoters based on combinatorial modules

For each genome, a list of each core promoter element's putative distribution range was determined based on the qualitative and quantitative measures as mentioned earlier. Custom perl scripts were used to determine the specific core element present in each promoter sequence of the genome. The set of core elements found to be present in a particular promoter sequence defined that promoter's combinatorial module. Though, with this approach one combinatorial module per promoter was obtained, however often a combinatorial module was found to be present in more than one promoter. Therefore, only the set of unique combinatorial modules was analyzed. To further determine prevalent CPEs contributing to the majority of the combinatorial modules, this dataset was partitioned with respect to the presence and absence of the TATA-box.

### Functional enrichment analysis of TATA, TATAless and coreless genes using gene ontologies

*A. thaliana* protein coding genes in TATA, TATA-less, and CPE-less categories were analyzed for gene ontology functional enrichment by using BiNGO [146]. BiNGO is a Cytoscape [147] plugin that calculates statistically over-represented ontology terms (GO molecular function and/or GO biological process) in a given set of foreground genes as compared to the background set of genes (entire genome). A hypergeometric distribution-based statistical enrichment method was used to assess functional enrichment in each category (p-value ≤0.01) and the Benjamini and Hochberg method [148] was used for multiple testing correction.

## Supporting Information

Figure S1 **The distribution of the number of transcripts with respect to 5′UTR length across dicots and monocots.** The panel A shows the distribution of 5′ UTR length in four dicots: *Arabidopsis thaliana* (Ath - solid navy blue), *Glycine max* (Gma-solid dark blue), *Populus trichocarpa* (Ptr –solid blue sapphire), and *Vitis vinifera* (Vvi -solid blue green). The panel B shows the distribution of 5′UTR length in four monocots: *Brachypodium distachyon* (Bdi-solid bronze yellow), *Oryza sativa ssp. japonica* (Osa-solid red), *Sorghum bicolor* (Sbi-solid bronze), and *Zea mays* (Zma -solid purple). X-axis shows bins of 5′ UTR length, where each bin is 10 base-pair long. Y-axis shows the number of transcripts.
(TIF)

Figure S2 **The GC content distribution across dicots and monocots.** X-axis shows the percentage GC across eight genomes whereas Y –axis shows the number of transcripts. GC percentage in monocots and dicots showed the demarcation of GC content distribution between dicots and monocots.
(TIF)

Figure S3 **The distribution of all known core promoter elements in dicot model (*Arabidopsis thaliana)*.** X-axis shows [−500,+500 with respect to TSS] promoter region that is binned into 20 base-pair bins, where each bin is represented by the bin-center. Y-axis shows the frequency distribution signal of the CPEs along the promoter with respect to TSS. CPEs include: MTE (golden), Inr (dark-grey), GC-box (black), CCAAT-box (purple), DPE (dark-green), BREu (brown), BREd (sky-blue), DCE-S1 (yellowish-green), XCPE1 (blue), TATA-box (red), MED-1 (light-grey), and Y-patch (green).
(TIF)

Figure S4 **The distribution of all known core promoter elements in monocot model (*Oryza sativa ssp. japonica)*.** X-axis shows [−500,+500 with respect to TSS] promoter region that is binned into 20 base-pair bins, where each bin is represented by the bin-center. Y-axis shows the frequency distribution signal of the CPEs along the promoter with respect to TSS. CPEs include: MTE (golden), Inr (dark-grey), GC-box (black), CCAAT-box (purple), DPE (dark-green), BREu (brown), BREd (sky-blue), DCE-S1 (yellowish-green), XCPE1 (blue), TATA-Box (red), MED-1 (light-grey), and Y-patch (green).
(TIF)

Figure S5 **The distribution of selected known core promoter elements in *Drosophila melanogaster*.** Genome-wide motif distribution profiles of core elements – TATA-box (solid red), Inr (solid blue), MTE (solid black), and DPE (solid green) in *D. melanogaster* genome showing positional conservation of these CPEs with respect to TSS. X-axis shows [−500,+500 with respect to TSS] promoter region that is binned into 20 base-pair bins, where each bin is represented by the bin-center. Y-axis shows the frequency distribution of the elements along the promoter with respect to TSS.
(TIF)

Table S1 **Genomic information of the eight plant species from Gramene.**
(XLSX)

Table S2 **Brief information on core promoter elements with sequence logo of the respective position weight matrix.**
(XLSX)

Table S3 **Position weight matrix specific score threshold cut off for each core promoter element for eight plant species.**
(XLSX)

Table S4 **Putative binding site distribution of the core promoter elements relative to transcription start sites based on positional and orthologous gene conservation across species.**
(XLSX)

Table S5 **Percentage distribution of the core promoter elements in eight plant genomes.**
(XLSX)

Table S6 **CPE combinatorial modules selected based on the positional distribution range across dicots.**
(XLSX)

Table S7 **CPE combinatorial modules selected based on the positional distribution range across monocots.**
(XLSX)

Table S8 **Prevalence of core promoter elements in combinatorial CPE modules in TATA(+) and TATA(−) promoter genes.**
(XLSX)

Table S9 **Functional annotation based on gene ontology molecular function enrichment of TATA, TATAless and Coreless genes.**
(XLSX)

Table S10 **Functional annotation based on gene ontology biological process enrichment of TATA, TATAless and Coreless genes.**
(XLSX)
