# Supplementary figures and images for: Genome-Wide Computational Prediction and Analysis of Core Promoter Elements across Plant Monocots and Dicots
Source: PLoS One. 2013 Oct 29;8(10):e79011. doi: 10.1371/journal.pone.0079011 (PMC3812177; doi:10.1371/journal.pone.0079011)

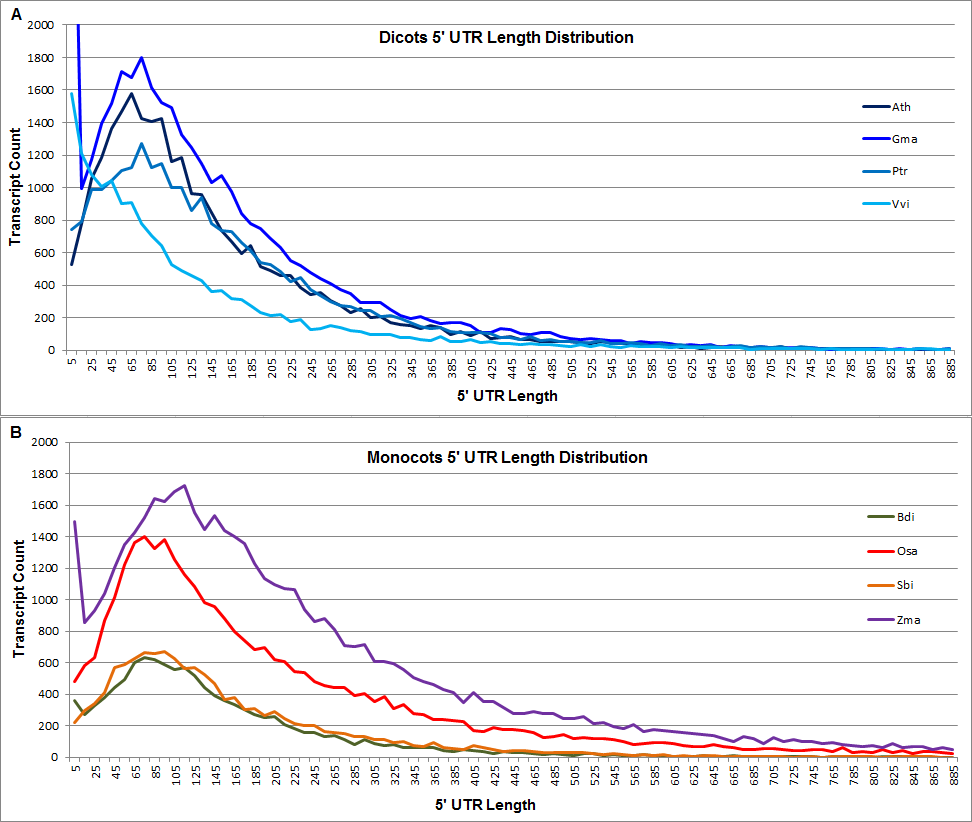

Supplement: Figure S1 — The distribution of the number of transcripts with respect to 5′UTR length across dicots and monocots. The panel A shows the distribution of 5′ UTR length in four dicots: Arabidopsis thaliana (Ath - solid navy blue), Glycine max (Gma-solid dark blue), Populus trichocarpa (Ptr –solid blue sapphire), and Vitis vinifera (Vvi -solid blue green). The panel B shows the distribution of 5′UTR length in four monocots: Brachypodium distachyon (Bdi-solid bronze yellow), Oryza sativa ssp. japonica (Osa-solid red), Sorghum bicolor (Sbi-solid bronze), and Zea mays (Zma -solid purple). X-axis shows bins of 5′ UTR length, where each bin is 10 base-pair long. Y-axis shows the number of transcripts. (TIF) [file pone.0079011.s001.tif]

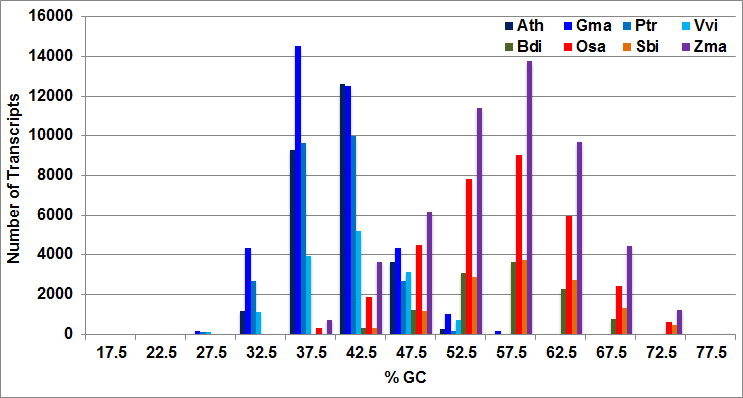

Supplement: Figure S2 — The GC content distribution across dicots and monocots. X-axis shows the percentage GC across eight genomes whereas Y –axis shows the number of transcripts. GC percentage in monocots and dicots showed the demarcation of GC content distribution between dicots and monocots. (TIF) [file pone.0079011.s002.tif]

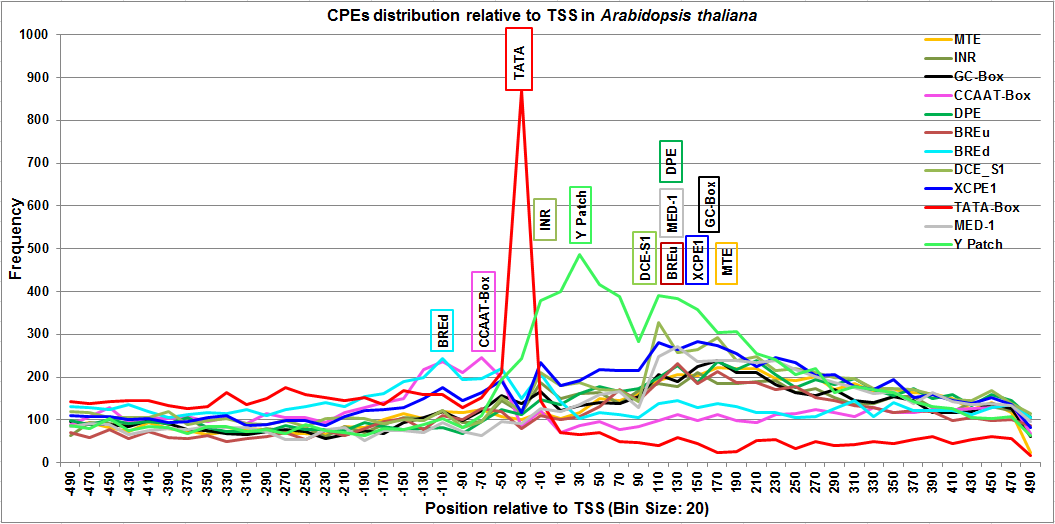

Supplement: Figure S3 — The distribution of all known core promoter elements in dicot model (Arabidopsis thaliana). X-axis shows [−500,+500 with respect to TSS] promoter region that is binned into 20 base-pair bins, where each bin is represented by the bin-center. Y-axis shows the frequency distribution signal of the CPEs along the promoter with respect to TSS. CPEs include: MTE (golden), Inr (dark-grey), GC-box (black), CCAAT-box (purple), DPE (dark-green), BREu (brown), BREd (sky-blue), DCE-S1 (yellowish-green), XCPE1 (blue), TATA-box (red), MED-1 (light-grey), and Y-patch (green). (TIF) [file pone.0079011.s003.tif]

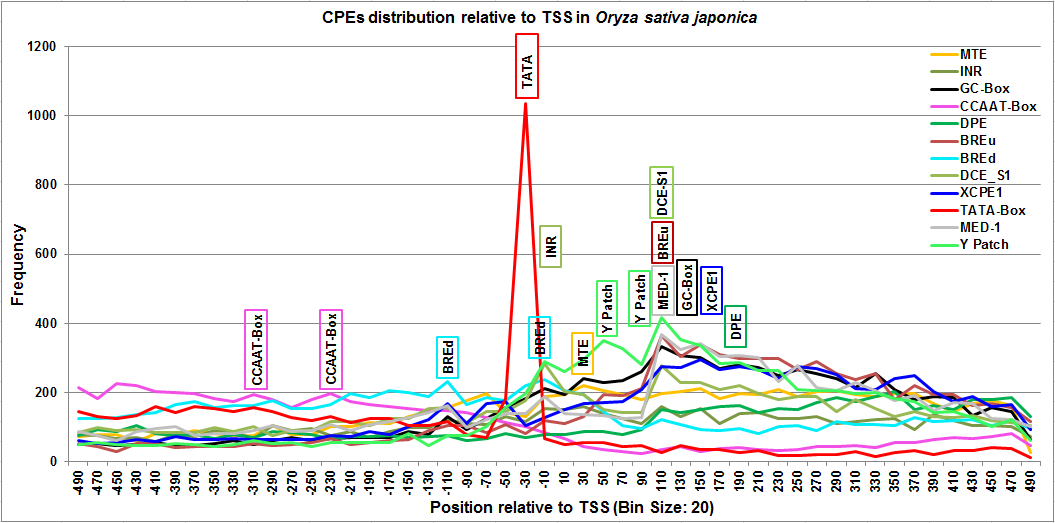

Supplement: Figure S4 — The distribution of all known core promoter elements in monocot model (Oryza sativa ssp. japonica). X-axis shows [−500,+500 with respect to TSS] promoter region that is binned into 20 base-pair bins, where each bin is represented by the bin-center. Y-axis shows the frequency distribution signal of the CPEs along the promoter with respect to TSS. CPEs include: MTE (golden), Inr (dark-grey), GC-box (black), CCAAT-box (purple), DPE (dark-green), BREu (brown), BREd (sky-blue), DCE-S1 (yellowish-green), XCPE1 (blue), TATA-Box (red), MED-1 (light-grey), and Y-patch (green). (TIF) [file pone.0079011.s004.tif]

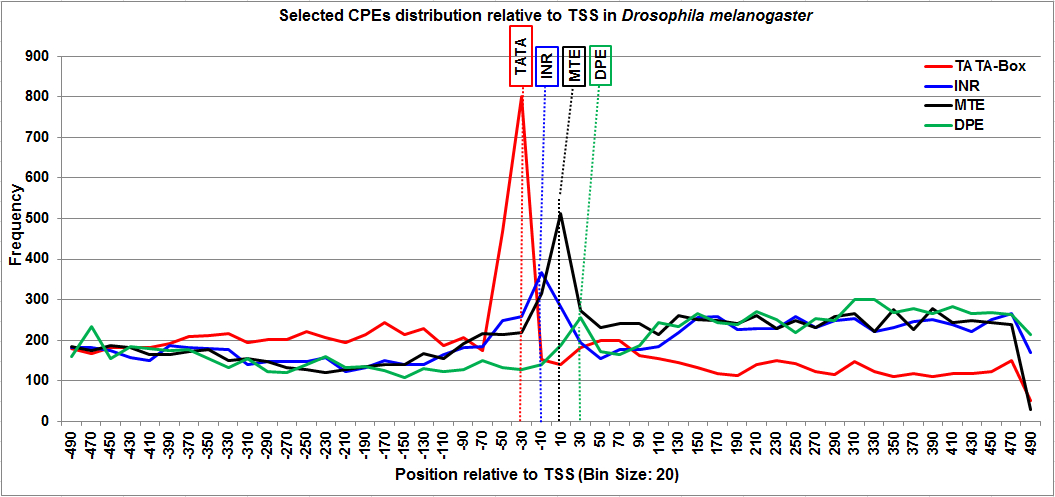

Supplement: Figure S5 — The distribution of selected known core promoter elements in Drosophila melanogaster. Genome-wide motif distribution profiles of core elements – TATA-box (solid red), Inr (solid blue), MTE (solid black), and DPE (solid green) in D. melanogaster genome showing positional conservation of these CPEs with respect to TSS. X-axis shows [−500,+500 with respect to TSS] promoter region that is binned into 20 base-pair bins, where each bin is represented by the bin-center. Y-axis shows the frequency distribution of the elements along the promoter with respect to TSS. (TIF) [file pone.0079011.s005.tif]
